# Drivers of *Solidago* species invasion in Central Europe—Case study in the landscape of the Carpathian Mountains and their foreground

**DOI:** 10.1002/ece3.7989

**Published:** 2021-08-10

**Authors:** Peliyagodage Chathura Dineth Perera, Tomasz H. Szymura, Adam Zając, Dominika Chmolowska, Magdalena Szymura

**Affiliations:** ^1^ Institute of Agroecology and Plant Production Wrocław University of Environmental and Life Sciences Wrocław Poland; ^2^ Department of Ecology, Biogeochemistry and Environmental Protection University of Wrocław Wrocław Poland; ^3^ Institute of Botany Faculty of Biology and Earth Sciences Jagiellonian University in Kraków Kraków Poland; ^4^ Institute of Systematics and Evolution of Animals Polish Academy of Sciences Kraków Poland

**Keywords:** alien plants, biological invasion, Boosted Regression Trees, drivers of invasion, PAB framework, *Solidago canadensis*, *Solidago gigantea*

## Abstract

**Aim:**

The invasion process is a complex, context‐dependent phenomenon; nevertheless, it can be described using the PAB framework. This framework encompasses the joint effect of propagule pressure (P), abiotic characteristics of the environment (A), and biotic characteristics of both the invader and recipient vegetation (B). We analyzed the effectiveness of proxies of PAB factors to explain the spatial pattern of *Solidago canadensis* and *S*. *gigantea* invasion using invasive species distribution models.

**Location:**

Carpathian Mountains and their foreground, Central Europe.

**Methods:**

The data on species presence or absence were from an atlas of neophyte distribution based on a 2 × 2 km grid, covering approximately 31,200 km^2^ (7,752 grid cells). Proxies of PAB factors, along with data on historical distribution of invaders, were used as explanatory variables in Boosted Regression Trees models to explain the distribution of invasive *Solidago*. The areas with potentially lower sampling effort were excluded from analysis based on a target species approach.

**Results:**

Proxies of the PAB factors helped to explain the distribution of both *S*. *canadensis* and *S*. *gigantea*. Distributions of both species were limited climatically because a mountain climate is not conducive to their growth; however, the *S*. *canadensis* distribution pattern was correlated with proxies of human pressure, whereas *S*. *gigantea* distribution was connected with environmental characteristics. The varied responses of species with regard to distance from their historical distribution sites indicated differences in their invasion drivers.

**Main conclusions:**

Proxies of PAB are helpful in the choice of explanatory variables as well as the ecological interpretation of species distribution models. The results underline that human activity can cause variation in the invasion of ecologically similar species.

## Graphical abstract

In this article, we analyzed the drives of distribution of two noxious invasive in Europe, Asia, and Australia plants. Besides the detection of main invasion drivers, we also discussed the findings to establish the best strategies of control of populations of these species at a large spatial extent. Because the studied Solidago species have a high environmental impact, on different continents, and the results based on large data set (7,800 squares 2 × 2 km) collected at a large spatial extent (3,200 km^2^), we believe that the results of this study will be interesting for a wide audience of scientist and managers.

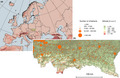

## 1 INTRODUCTION

Biodiversity and the function of ecosystems are threatened by global change drivers such as changes in land use and climate, as well as biological invasions (Linders et al., 2019; Sala et al., 2000). Invasive species alter a wide range of ecosystem services, including provisioning, regulation, and cultural and supporting functions, and they are particularly hazardous for biodiversity maintenance, human welfare, and the economy (Charles & Dukes, 2007; Chytrý et al., 2009; Hejda et al., 2009; Pejchar & Mooney, 2009; Vilà & Ibáñez, 2011). Globalization (e.g., international trade and travel) and climate change (e.g., global warming, droughts, and floods) can interact, which can in turn increase the level of biological invasions (Catford et al., 2009; Le Maitre et al., 2004; Pino et al., 2005; Seebens et al., 2015). As the total number of invasive species increases, some sites may host several alien species (Kuebbing & Nuñez, 2015).

The invasion process is a complex phenomenon, driven by numerous interacting processes, and the effect of this interaction is highly contingent on the context (Chamberlain et al., 2014; Frost et al., 2019). Consequently, drivers of plant invasion can vary depending on the specific region and habitat (Taylor et al., 2016). Nevertheless, invasions have a common pattern, which can be summarized as the joint effect of propagule pressure, abiotic characteristics of the environment, and biotic characteristics of both the invader and recipient vegetation (Catford et al., 2009), the so‐called PAB framework. Propagule pressure (P) includes dispersal and geographical constraints, while abiotic characteristics (A) comprise environmental and habitat constraints and biotic characteristics (B) describe the internal dynamics of the vegetation and community interactions (Catford et al., 2009). All these factors operate at different spatial scales (Czarniecka‐Wiera et al., 2020; Milbau et al., 2009) and are influenced by human activity (Essl et al., 2011). In practice, different indices can be applied as proxies of propagule pressure and abiotic and biotic conditions in modeling plant invasion process (Bazzichetto et al., 2018; Beaury et al., 2020; Chytrý et al., 2008; Szymura et al., 2018).

Related to the propagule pressure, the biological invasion correlates with many anthropogenic factors, such as density of the communication network, percentage of urban areas, gardening, and the fragmentation of natural habitats. Such factors can serve as a proxy of propagule pressure (Foxcroft et al., 2011; Pollnac et al., 2012; Štajerová et al., 2017; Szymura et al., 2018; Vilà & Ibáñez, 2011). In addition, economic and demographic variables reflect the intensity of human activities; therefore, socioeconomic factors such as gross domestic production and human population density can be important in predicting the invasion level (Essl et al., 2011; Hulme, 2017; Pino et al., 2005; Pyšek & Richardson, 2010) because they correlate with trade intensity and communication network density (Hulme, 2009). Among the abiotic interactions with the greatest impact on a large spatial scale (continental, regional), climate is considered the most critical in limiting the geographic distribution of species (Hulme, 2017; Thuiller et al., 2007). In terms of resource availability, invasive species usually prefer productive habitats where they are able to achieve competitive dominance (Czarniecka‐Wiera et al., 2020; Peltzer et al., 2016; Perkins et al., 2011). In addition, environments with high variability in resource availability, resulting from periodic external supply (e.g., surface runoff) or destruction of local vegetation that previously used the resources (e.g., human disturbances, abandonment of agricultural crops), are more susceptible to invasions than habitats with stable availability of resources (Davis et al., 2000; Kulmatiski et al., 2006; Rejmánek, 1989). Given the biotic characteristics of the invader and receipt communities, the limiting similarity hypothesis proposes that the invasion by alien species will be successful if the native species of the recipient community differ from the invader in terms of functional traits and resource requirements (MacArthur & Levins, 1967), which decreases competition for resources (Funk et al., 2008). Thus, the functional traits of the invader should not overlap with traits of native plants occurring in the invaded community, which will allow it to occupy an empty niche and successfully invade the community (Funk et al., 2008; Hejda & de Bello, 2013). Because some sites can be invaded by several species simultaneously, determining the interaction between invaders is critical for understanding their distribution (Kuebbing & Nuñez, 2015). For example, the local species assemblage can be driven by a priority effect, and the effect is particularly strong when interacting species have similar use of resources (Vannette & Fukami, 2014). In practice, the abundance and composition of invasive species are also related to landscape characteristics (e.g., habitat fragmentation, patch size, shape, and connections), habitat type, land use, and the composition of the surrounding landscape because these factors correlate with propagule pressure and habitat quality and availability (Basnou et al., 2015; Chytrý et al., 2009; González‐Moreno et al., 2013; Štajerová et al., 2017; Szymura et al., 2016).

Because of the complexity of biological invasion, better understanding of the underlying factors and their management is challenging. As tools for obtaining reliable and repeatable information for biological analyses as well as nature conservation and management of the invaders, invasive species distribution models (iSDMs) are considered useful (Lozano et al., 2020; Zurell et al., 2020). Modeling species’ environmental requirements and mapping their distributions through space and time help to identify the main introduction pathways and secondary spread and the areas and land use types that are more prone to invasion. These various threads could be woven into a strategy of prevention and elimination of invasive plant species on a regional scale (Lozano et al., 2020). Despite their deficiencies (e.g., problematic species‐environment equilibrium; Gallien et al., 2012; Hattab et al., 2017), iSDMs are still useful in the face of accelerating global changes and data deficiencies, as well as limited research funding (Yates et al., 2018). The PAB approach, despite its obvious advantages for selection of explanatory variables and model results interpretation, has rarely been used within an invasive species distribution modeling framework (but see Bazzichetto et al., 2018; Czarniecka‐Wiera et al., 2020; Lozano et al., 2020).

Goldenrod species from North America represent successful invaders in Europe, Asia, Australia, and New Zealand (Gusev, 2015; Szymura & Szymura, 2013; Ye et al., 2019; Zhang & Wan, 2017). In Central Europe, two invasive *Solidago* species occur, *S*. *gigantea* Aiton (giant goldenrod) and *S*. *canadensis* L. (Canadian goldenrod). Due to their high environmental impact, wide range of distribution, and locally high abundance, invasive *Solidago* species have to be controlled in Europe (Fenesi et al., 2015; Sheppard et al., 2006; Skórka et al., 2010). They have been proposed for addition to the list of hazardous alien species that threaten ecosystems, habitats, or other species in European Union countries (CABI, 2018; EPPO, 2020; Tokarska‐Guzik et al., 2015). Unfortunately, the eradication of widely established invasive plant species, such as *Solidago*, is not feasible. The management strategies need to integrate different options that account for the distribution and abundance of the invader, its environmental niche, and the areas that are likely to experience high impacts (Nagy et al., 2020; Shiferaw et al., 2019; Woodford et al., 2016). Management needs to consider intrinsic factors related to the biology and ecology of the invader, as well as extrinsic environmental factors, such as dispersal vectors and invasion pathways (Shiferaw et al., 2019).

*Solidago canadensis* and *S*. *gigantea* differ with regard to ecological niche in their native range (Johnson, 1995; Werner et al., 1980) and the time of introduction into Europe (Tokarska‐Guzik, 2005). However, previous studies suggest that these two species do not differ regarding their habitat preferences in Central Europe, and observed differences in their spatial distribution patterns emerge from historical contingency and limitation in long‐range dispersal (Szymura & Szymura, 2016). The two *Solidago* species occupy different areas and rarely form mixed‐species stands (Szymura & Szymura, 2016). In this study, we aimed to find the main drivers of *Solidago* species’ invasion at a regional scale, using a species distribution model and applying the PAB framework for selection of adequate explanatory variables and for ecological interpretation of the models. The distribution models can be used for mapping of invasion probability at a regional level to facilitate invasion control at a macroecological scale.

## 2 MATERIALS AND METHODS

### 2.1 Studied species

Goldenrod species are hemicryptophytes (shoots are annual and newly sprout each spring) with rhizomes; they are insect pollinated and self‐incompatible, with inflorescences forming at the top of each shoot which can produce up to 10,000–20,000 wind‐dispersed seeds per one ramet (Bielecka et al., 2017; Guzikowa & Maycock, 1986; Moran et al., 2017; Schmid et al., 1988). The seeds of *S*. *canadensis* and *S*. *gigantea* have a high germination percentage (Weber, 2000; Weber & Jakobs, 2005), but in dense, well‐established *Solidago* stands, seed germination and seedling emergence are exceptional. The clone size increases via horizontal rhizomes, and the death of an established genet is a rare event (Meyer & Schmid, 1999a, 1999b).

The native habitats of *S*. *canadensis* are tall‐grass prairies, infrequently grazed pastures, abandoned farmlands, roadsides, and waste areas in North America (Johnson, 1995; Werner et al., 1980). *Solidago gigantea* prefers moist habitats, such as woods, stream edges, and woodland borders (Johnson, 1995). In Europe, *S*. *gigantea* and *S*. *canadensis* occupy similar habitats and prefer fallow lands and ruderal habitats on moist to mesic sites, such as abandoned farmlands, scrub, roadsides, forest edges, grasslands, wetlands, and riversides (Szymura & Szymura, 2013, 2016). Invasive goldenrods are highly competitive for nutrients, water, and space, and they release allelopathic compounds that inhibit growth of other plants (Gusev, 2015; Ledger et al., 2015; Werner et al., 1980; Zhang & Wan, 2017). Due to prolific vegetative propagation, they form dense stands and decrease the biodiversity of plants (Chmura et al., 2016; Ye et al., 2019; Zhang & Wan, 2017); arthropods (de Groot et al., 2007), including pollinators (e.g., wild bees, hoverflies, and butterflies) (Lenda et al., 2020; Moroń et al., 2009, 2021) and ants (Kajzer‐Bonk et al., 2016; Lenda et al., 2013); and birds (Skórka et al., 2010).

*Solidago canadensis* was the first alien *Solidago* species recorded in Europe, in 1648, while *S*. *gigantea* was first recorded in 1758. The species were found in the territory of Poland about 100 years later, *S*. *gigantea* in 1853 and *S*. *canadensis* in 1872 (Tokarska‐Guzik, 2005). After *S*. *canadensis* and *S*. *gigantea* were introduced into botanical gardens, they were distributed among gardeners. The plants were attractive and easy to grow as ornamental plants, and they were useful for beekeepers (Guzikowa & Maycock, 1986; Roháčová & Drozd, 2009; Weber, 1997; Zihare & Blumberga, 2017). Recently, *Solidago* species have become widely distributed throughout Poland. According to the stages of invasion (Blackburn et al., 2011), *S*. *canadensis* and *S*. *gigantea* are now fully invasive species, with individuals dispersing, surviving, and reproducing at multiple sites in a wide variation of habitats over an extensive spatial area (E category).

### 2.2 Study area and species distribution data

**FIGURE 1:**
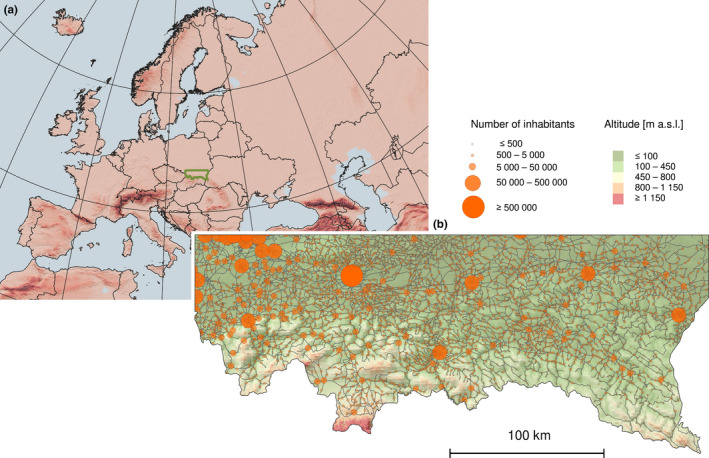
The study region location (green) on a background of land relief (a), and distribution of communication network and settlements on the background of altitude within the study region (b)

The study area comprises approximately 31,200 km^2^ in the southeast part of Poland, which extends from latitude 50.2° to 49°N and longitude from 19° to 23°E (Figure 1). This area is diversified due to environmental conditions mostly shaped by the altitude ranging from 160 to 2,503 m a.s.l. Additional factors underlying diversity are correlated with climate, land use systems, land relief, and human population density. In the northern part, the lowland areas are used for agriculture and the foothills are dominated by forests, and the southern part has high mountains with alpine vegetation. In addition to the north–south altitudinal gradient, there is also a climatic gradient of continentality, with higher temperature range in the eastern part of the study region (Szabo‐Takacs et al., 2015) which, in the studied region, correlated strongly with decreasing eastward precipitation (Appendix S4, Table S3). The study area includes a densely populated industrial landscape (Silesia), urban agglomerations (largest city Kraków), and moderately populated agricultural areas, as well as sparsely populated areas in the mountains. The detailed characteristics of the study area (climate, topography, land use structure, and human population density) were previously described by Szymura et al. (2018).

The data on distribution of the studied *Solidago* species were obtained from the atlas Distribution of Kenophytes in the Polish Carpathians and their Foreland (Zając & Zając, 2015), which shows maps of species presence or absence in a 2 × 2 km grid in the Polish part of the Carpathian Mountains and their foreland, Central Europe. The fieldwork designed for the purpose of compiling the atlas was based on a survey of flora in particular regions (e.g., mountain ranges, particular towns, and surrounding areas) and exploration focused exclusively on neophytes in given regions. These observations were supplemented with additional data from phytosociological relevés, herbarium records, and published materials. The fieldwork was carried out by several dozen professional botanists as well as graduate students, focusing on a predefined 2 × 2 km grid for sampling (Zając A., personal information). This work represents a “survey” type of data, according to Elith et al. (2020) nomenclature. Such data, with true absence records, enable species distribution models to be less biased and to perform better, compared with presence‐only records, the “collection” data type (Barbet‐Massin et al., 2012; Elith et al., 2020). This distinction is of particular importance for examination of wide‐ranging and tolerant species (Brotons et al., 2004). To reduce the possible effect of lower sampling effort in some regions (Bailey et al., 2017; Yang et al., 2013), the potentially undersampled squares were excluded from modeling. For this purpose, we used a “target group approach” (Chapman et al., 2019; Phillips et al., 2009) and a previously established model which explains neophyte richness (the “target group” in this case) as a function of environmental and socioeconomic variables in the studied region (Szymura et al., 2018). We assumed that the squares with the highest negative model residuals (i.e., squares where recorded neophyte richness was much lower than predicted by the model) indicated potentially undersampled regions. After preliminary testing, we decided to exclude from modeling 25% of squares (1950 squares) with the highest negative residual values and simultaneously without any invasive *Solidago* records (for details of this calculation see Appendix S1).

### 2.3 Explanatory variables and statistical analysis

We prepared a data set of environmental variables that can be considered as proxies of propagule pressure, abiotic environment, and biotic characteristics, based on the PAB framework (Catford et al., 2009; Table 1). These proxies were chosen based on the results of previous study on *Solidago* (Szymura et al., 2016) and the most influential drivers of neophytes in the region (Szymura et al., 2018).

**TABLE 1:** Explanatory variables selected for modeling invasive *Solidago* distribution. Variables in bold type were used in the final model, and the remaining variables were excluded from further analysis due to collinearity

Explanatory variable	Abbreviation	Probable sphere of PAB framework
**Communication routes (railways and roads) density**	***communication***	**P**
**Shannon's diversity index of landscape**	***SHDI***	**B**
*Urban area percentage*	*urban*	P
**Cropland area percentage**	***cropland***	**B**
*Forest area percentage*	*forest*	B
**Human population density**	***density***	**P**
**Income per capita**	***income***	**P**
*Topographic roughness index*	*TRI*	A
**Topographic position index**	***TPI***	**A**
**Average annual temperature**	***temperature***	**A**
**Topographic wetness index**	***TWI***	**A**
**Temperature seasonality**	***Ts***	**A**
*Annual sum of precipitation*	*precipitation*	A
**CaCO_3_ content**	***Ca***	**A**
**K content**	***K***	**A**
*N content*	*N*	A
*P content*	*P*	A
**pH in H_2_O**	***pH***	**A**
**Distance to nearest introduction site *S. canadensis***	***distance_S.can***	**P**
**Distance to nearest introduction site *S. gigantea***	***distance_S.gig***	**P**
**Presence of competing *Solidago* species** ^a^	***competitor***	**B**

^a^ Presence of one invasive *Solidago* species in the same 2 × 2 km square was considered as an explanatory variable for the other; that is, in model for *S*. *canadensis*, its presence explained the presence of *S*. *gigantea* and vice versa.

The anthropogenic variables were derived from CORINE 2012 database (*urban*), the Central Statistical Office of Poland (*income*), and Statistics Poland (*density*). The income (as an estimator of wealth) is directly correlated with trade intensity and thus reflects the potential to alien species propagule transportation by trade or accidentally (Hulme, 2009; Pyšek et al., 2010). The length of communication routes (*communication*) was obtained from the Polish Geographical Objects Database (BDOO). The other data were calculated from the CORINE 2012 database (*cropland, forest, SHDI*). A Digital Elevation Model for Europe (EU‐DEM) was used to calculate the topographic metrics (*TPI* and *TWI*). Maps prepared by Ballabio et al. (2019) using data from Land Use and Cover Area frame Survey (LUCAS) were used to calculate soil characteristics (content of *N*, *P*, *K*, and soil *pH*). The climate data (*precipitation*, *temperature*) were derived from a climatic model developed by Hijmans et al. (2005). Before the analyses, the Pearson correlations between each pair of explanatory variables were checked. If the coefficient exceeded 0.7, one of the correlated variables was eliminated to avoid collinearity (Dormann et al., 2013). For details, see Appendix S3 Table S2. The average values of the variables were calculated for each 2 × 2 km grid cell acquired from Zając and Zając (2015), and the landscape diversity (SHDI) was expressed by Shannon's diversity index.

Maps showing the historical distribution of goldenrods before their spreading phase (Tokarska‐Guzik, 2005) were used to calculate the distances from a focal 2 × 2 km square to the nearest site of goldenrod introduction in the 1950s (*distance*, for details see Appendix S3, Map S2). To check whether the presence of one *Solidago* species in a 2 × 2 km square explained the presence of the second species (possible priority effect), the data on distribution of the potential competitor were used as an explanatory variable (*competitor*). All the calculations and map handlings were done using QGIS, SAGA GIS, and FRAGSTAT software.

Goldenrod species spatial pattern of distribution was modeled using a boosted regression trees (BRT) technique (De’Ath, 2007; De’Ath & Fabricius, 2000) employing packages gbm (Greenwell et al., 2020), dismo (Hijmans et al., 2020), and Biomod2 (Thuiller et al., 2020) in the R environment. After initial examinations, the BRT settings were applied: tree complexity, 5; bag fraction, 0.5; learning rate, 0.001; and cross‐validation, 10 fold. The optimal number of trees was 3,900 for *S*. *canadensis* and 3,850 for *S*. *gigantea*. Models for each species were constructed using all explanatory variables and then simplified to obtain the parsimonious model. The BRT modeling and simplification of models were done based on Elith et al. (2008) suggestions. Then, the modeling, using the tuned model parameters and a minimal set of explanatory variables, was performed in Biomod2 package with spatially blocked cross‐validation (Valavi et al., 2019). We applied 5‐fold cross‐validation, using spatial blocks constructed based on 10 × 10 km squares for *S*. *canadensis* and 20 × 20 km squares for *S. gigantea*. The sizes of the squares were chosen based on spatial autocorrelation of raw distribution data (Roberts et al., 2017), and the blocks were constructed using BlockCV package within the R environment (Valavi et al., 2019). For details of this approach, see the Appendix S1. The performance of the models was evaluated using area under the receiver operating characteristic curve (AUC). Following qualitative descriptions, an AUC value in the range of 0.7–0.8 can be considered a good prediction, 0.8–0.9 as a very good prediction, and above 0.9 as an excellent prediction (Šimundić, 2009). The ecological interpretation of the model relies on the drawing response curves for each explanatory variable (Elith et al., 2005) and calculation of relative influences of explanatory variables. The relative influence is calculated after model training (calibration) and prediction making. Then, values of one of the variables are randomized, and a new prediction is made. The correlation between this new prediction and the standard prediction is calculated and is considered as an estimation of the variable importance in the model: A high correlation score between the two predictions shows that the randomized variable has little influence on making a prediction and is not considered important for the model in its prediction. In contrast, a low correlation means a significant difference in the prediction making, showing that the variable is important for the model. The variable importance is calculated as 1‐correlation, repeated for each variable independently and for each spatially blocked cross‐validation run (Thuiller et al., 2012). Eventually, maps of projected *S*. *canadensis* and *S*. *gigantea* probability of occurrence were drawn (Figure 5). The probability of species presence in a given 2 × 2 km square was modeled for particular spatially blocked cross‐validation runs and averaged, employing the “projection” function in the Biomod2 package. Additionally, maps of squares projected to be suitable for invaders and not colonized yet were produced for conservation purposes (Appendix S7, Map S4).

## 3 RESULTS

Goldenrod species were observed in 60.5% of the squares (in 3,544 out of 5,850 finally examined squares). *Solidago gigantea* was the most frequent species (53.1%, 3,107 squares) followed by *S*. *canadensis* (21.4%, 1,255 squares).

**FIGURE 2:**
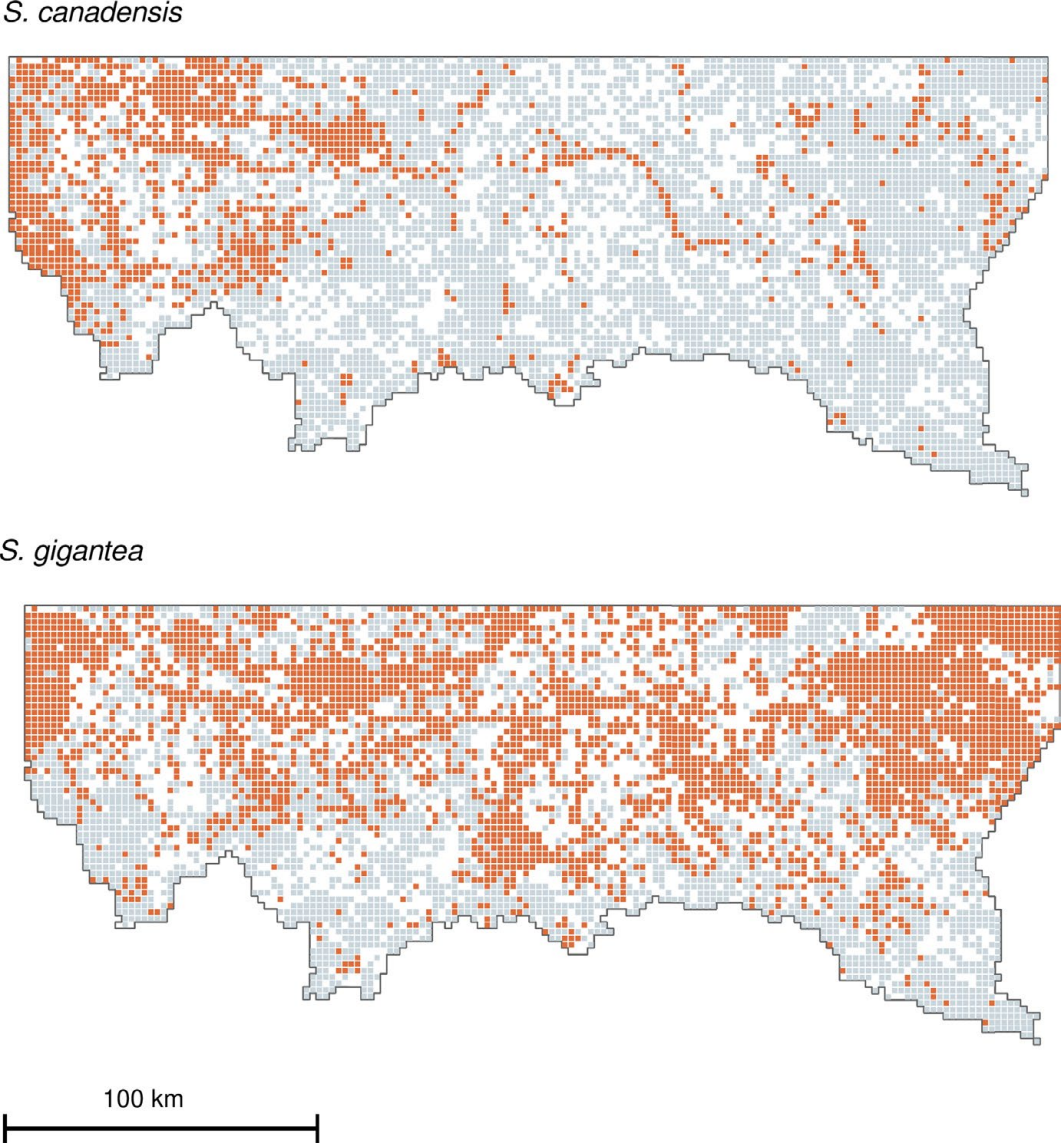
Distribution of invasive *Solidago* species (orange color) in studied region. The light gray color show distribution of squares with confirmed *Solidago* absence. Squares excluded from analysis, are not shown (left blank)

*Solidago gigantea* localities were widespread throughout almost the entire area, aside from the higher altitudes in the southern part of the study region. The *S*. *canadensis* was concentrated in the western part of the study area, while being sporadically dispersed in the eastern part and also avoiding the southern fragment with higher altitudes (Figure 2).

**FIGURE 3:**
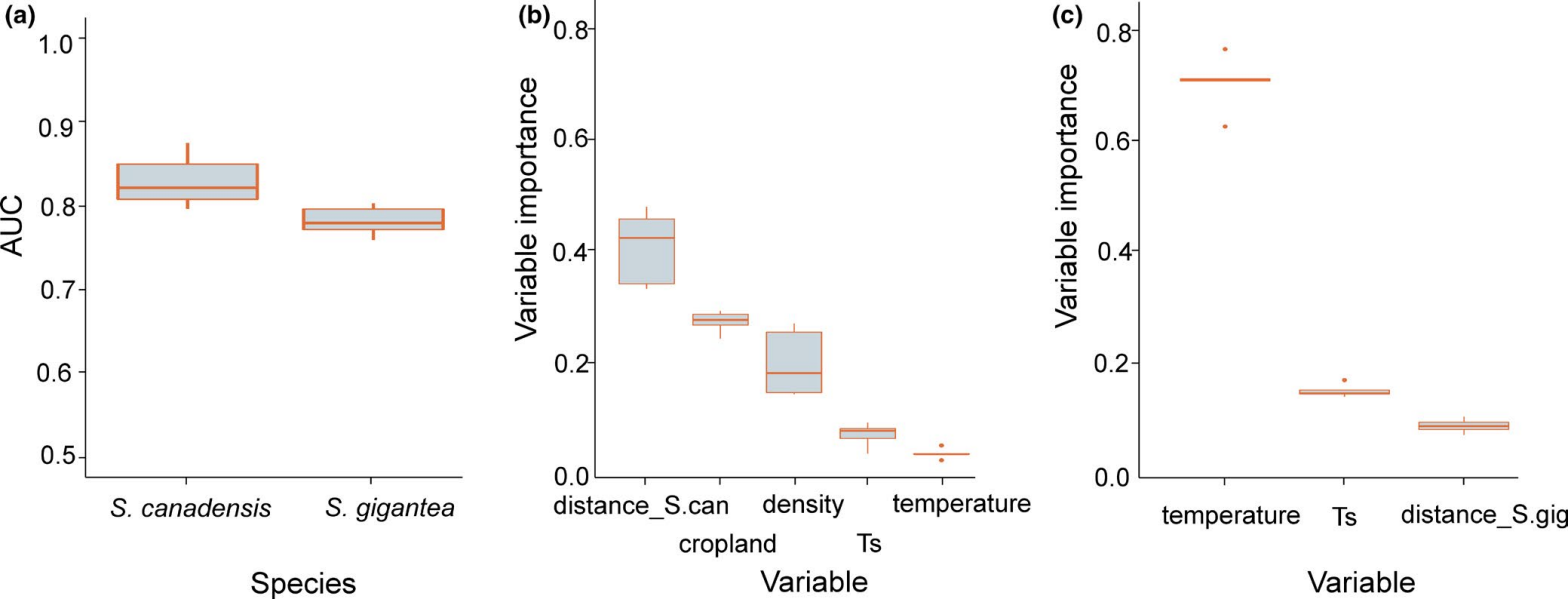
The values of area under curve (AUC) for simplified models of *S*. *canadensis* and *S*. *gigantea* distributions (a), and variable importance for each variable involved in the simplified models of (b) *S*. *canadensis* and (c) *S*. *gigantea*. The boxplot shows the results of runs in the spatially blocked, 5‐fold cross‐validation. The values of variable importance close to 1 indicate high variable importance to the model, while those close to 0 have low importance. Abbreviations: *cropland*, cropland area percentage; *density*, human population density; *temperature*, average annual temperature; *Ts*, temperature seasonality; *distance_S.can*, distance to nearest introduction site *S*. *canadensis*; *distance_S.gig*, distance to nearest introduction site *S*. *gigantea*

The average value of AUC was 0.836 for *S*. *candensis* and 0.786 for *S*. *gigantea*. Despite some differences in model evaluations of particular spatially blocked folds, the models for *S*. *canadensis* generally performed better than those for *S*. *gigantea* (Figure 3a). The parsimonious (simplified) model for *S*. *canadensis* relied on a higher number of explanatory variables than those for *S*. *gigantea*.

Both species reacted to climatic conditions, expressed by the annual average temperature (*temperature*) and temperature seasonality (*Ts*), as well as the distance from the initial introduction sites (*distance*) (Figure 3b,c). Moreover, the spatial pattern of distribution of *S*. *canadensis* was also explained by anthropogenic factors, such as human population density (*density*) as well as the percentage of agricultural lands (*cropland*). The full list of all variables included in the final models, along with their relative influence, is shown in Figure 3b,c.

**FIGURE 4:**
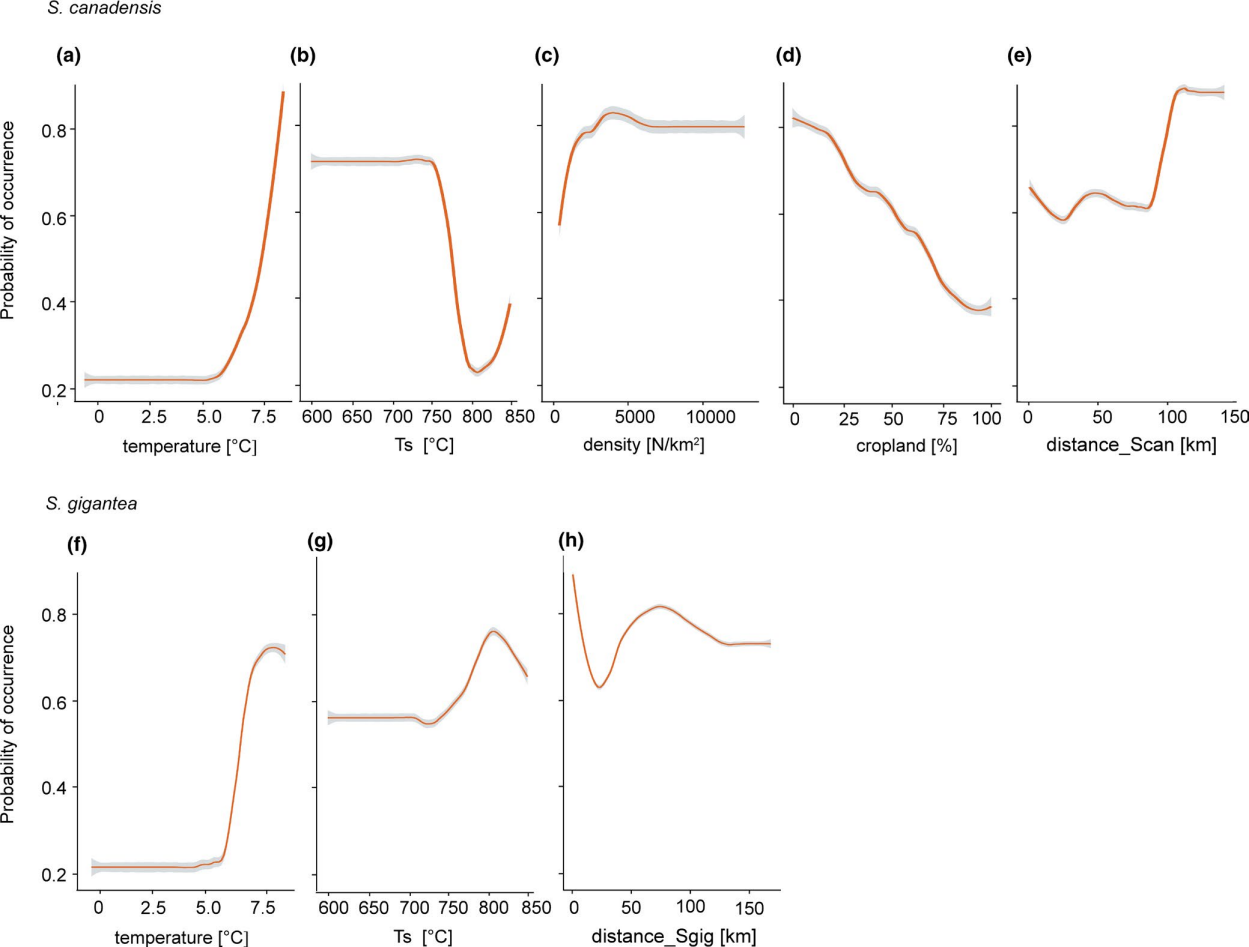
The modeled responses of *Solidago* species for particular environmental variables. The shape of the response was modeled using the evaluation strips method (Elith et al., 2005), with spatially blocked, 5‐fold cross‐validation. The graphs are sorted according to decreasing value of variables’ importance, upper panel for *S*. *canadensis*, lower for *S*. *gigantea*. Abbreviations: *cropland*, cropland area percentage; *density*, human population density; *temperature*, average annual temperature; *Ts*, temperature seasonality; *distance_S.can*, distance to nearest introduction site *S*. *canadensis*; *distance_S.gig*, distance to nearest introduction site *S*. *gigantea*

The modeled response of species on particular variables is shown in Figure 4. The distribution of both species was climatically limited, with the species being unlikely to occur in regions with an average annual temperature below 5.5°C. The probability of *S*. *canadensis* occurrence increased with human population density (*density*) (Figure 4), as well as distance from its introduction site (*distance_S.can*), with squares placed 100 km distant from the initial sites of introduction having the highest probability. The distribution of *S*. *gigantea* was also correlated with the pattern of its initial introduction (*distance S. gig*), and the probability of its occurrence generally decreased with the distance (Figure 4), reaching the lowest value at about 40 km and fluctuating above it.

**FIGURE 5:**
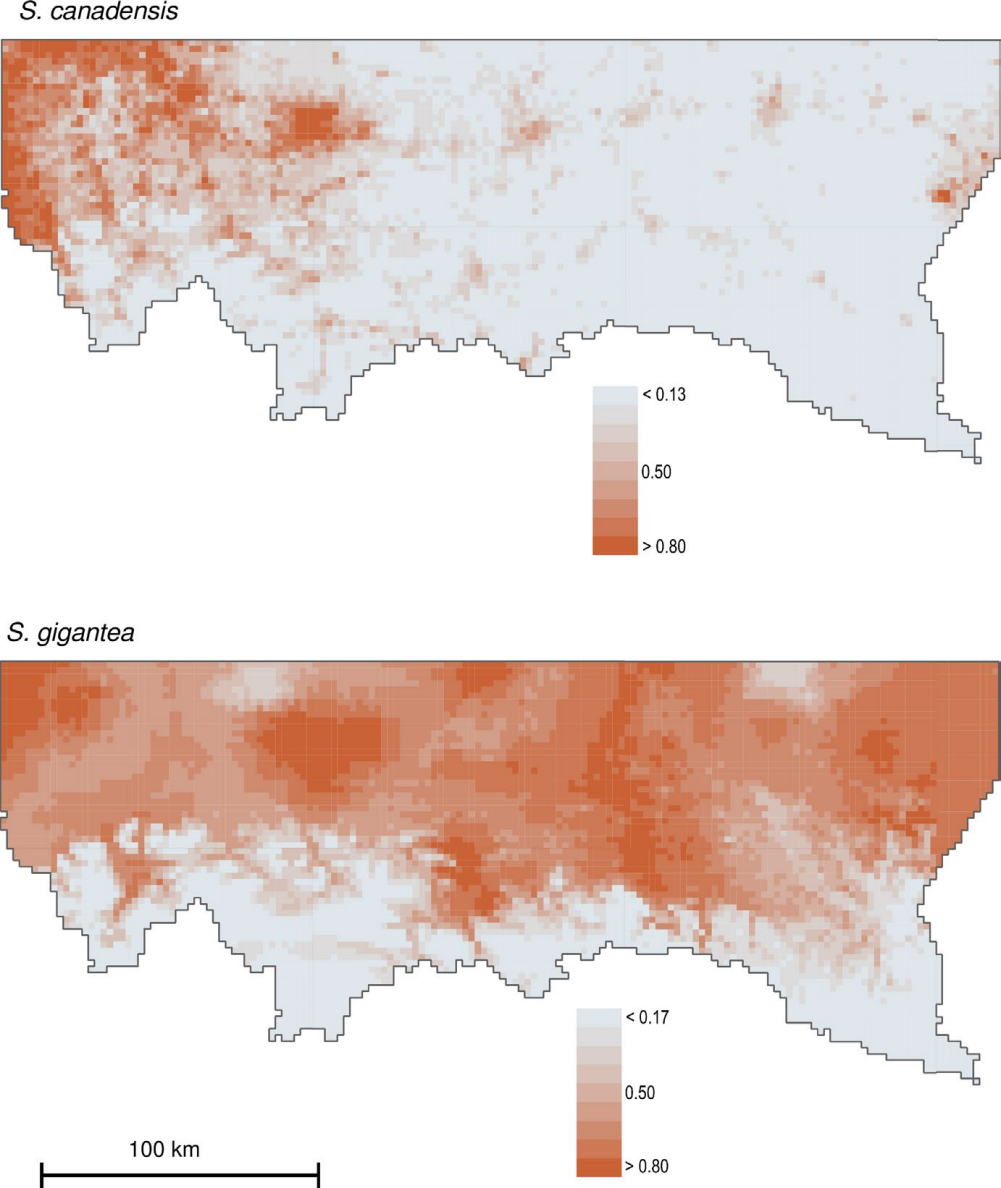
The projected probability of presence of the invasive *Solidago* species. The optimal cutoff value was 0.205 for *S*. *canadensis* and 0.539 for *S*. *gigantea*

The results of the projections are presented in Figure 5. The average cutoff values, calculated based on the AUC values, were 0.205 for *S*. *canadensis* and 0.539 for *S*. *gigantea*. In a comparison of the observed distribution with the model's prediction, the number of squares suitable for the invaders and not colonized yet (including the undersampled squares excluded from the analyses) increased by 45% (1,255 squares with presence versus. 2,293 predicted) for *S*. *canadensis* and 36% (3,107 squares with presence vs. 4,897 predicted) in the case of *S*. *gigantea*. For detailed maps see Appendix S7, Map S4.

## 4 DISCUSSION

The model's performance in interpreting the AUC values (Šimundić, 2009) should be considered as good for *S*. *gigantea* and very good for *S*. *canadensis*, despite the relatively limited number of explanatory variables retained after the model's simplification. Moreover, in the case of species with broad environmental tolerance, such as the studied *Solidago*, the model's performance is usually lower than it is in comparing with specialist species, both plants and animals (Guisan et al., 2007; Regos et al., 2019). The model's performance is improved by variables that can be interpreted as proxies of P, A, and B factors (see discussion below); however, the importance of the variables differed considerably between particular P, A, and B factors, as well as species studied.

### 4.1 Ecological interpretation of the models

#### 4.1.1 Propagule pressure

The recent distributions of examined species were correlated with initial patterns of their introductions in the 1950s. Quite surprisingly, the two species revealed an opposite relationship to these historical patterns. In the case of *S*. *gigantea*, the pattern was rather simple and intuitive: The probability was highest in squares closer to the sites of initial distribution. However, *S*. *canadensis* quite surprisingly was the most likely to occur in squares 100 km from the initial sites of introduction. These results suggest different mechanisms of long‐range dispersals, not related to biological issues, since their seeds, dispersal mechanism, and flowering time are similar (Weber, 2000; Weber & Jakobs, 2005).

Recently, *S*. *canadensis* was considered to have a higher ornamental value (because of larger size, bigger inflorescences, and clump occurrence) than *S*. *gigantea*. As a result, it is offered by garden shops, but *S*. *gigantea* is not (Szymura M. personal observations, data from internet shops offering ornamental plants). A similar pattern of trade has been described in Estonia, Central Europe, where only *S*. *canadensis* is offered in markets (Ööpik et al., 2013). Moreover, the honey from *S*. *canadensis* has recently been promoted on social media, without supporting scientific data, as a “superfood” with healing properties. This claim could encourage beekeepers to produce goldenrod honey, which would lead to further spread of *S*. *canadensis* and exacerbate its existing negative environmental impact (Lenda et al., 2020). Thus, it could be assumed that the long‐range dispersal of *S*. *canadensis* is recently enhanced by humans.

The distribution of *S*. *canadensis* is positively correlated with human population density. This straightforward correlation breaks if the population density exceeds 5,000 ind km^−2^. This happened in a few of the most densely inhabited squares, representing strict city centers. It was generally found that the plant species richness in areas with moderate levels of urbanization (e.g., suburban areas) exceeded the richness recorded in nonurbanized areas as well as in central, urban core areas (McKinney, 2008). The lack of a further increase in alien species richness in strict city centers, despite the high propagule pressure, was explained by the loss of suitable areas for plants (McKinney, 2008). Such generally limited neophytes’ richness caused by population density has previously been shown for this region (Szymura et al., 2018).

The results of the modeling support the assumption that recent *S*. *gigantea* dispersal has occurred mostly spontaneously without any human aid, while *S*. *canadensis* dispersal is still related to human presence and, additionally, intentional transport over longer distances via, for example, Internet commerce (Lenda et al., 2014). This pattern is partially related to longer invasion history of *S*. *gigantea* comparing to *S*. *canadensis*, with caused that the studied region is exposed to *S*. *gigantea* seed for a longer time.

#### 4.1.2 Abiotic factors

The variables representing abiotic environment (A) are the most important for model performance for both species; however, the impact of these variables was more pronounced in the case of *S*. *gigantea*, compared with *S*. *canadensis*.

The distribution of both species was restricted climatically, and their presence was unlikely in areas with an average yearly temperature below approximately 5.5°C. The temperature corresponds with the altitudinal zonation of vegetation in the studied region and relates to a lower limit of the montane zone, starting from an altitude of approximately 600–850 m a.s.l. in the studied region. The negative effect of cold climate on the distribution of both *Solidago* species studied is in accordance with studies examining their potential distribution in Europe, which indicated that northern Europe as a region is outside their climatic requirements (Weber, 2001). Although both species can be observed sporadically at higher altitudes, their typical upper limit is 1,200 m a.s.l. (Moran et al., 2017; Weber & Jakobs, 2005). In the case of *S*. *gigantea*, positive correlations have been found between the mean temperature and growth parameters, and high spring temperatures (above 24°C) are advantageous for germination (for review, see Weber & Jakobs, 2005). *Solidago canadensis* plants are taller at lower attitudes, and at higher altitudes, they are not able to develop seeds because of the limited length of the vegetation period (Moran et al., 2017). It should be noted that the data referred to here regarding altitude come from the central Alps, while the climate in the Carpathian Mountains is more severe; therefore, the upper limits of the vegetation zones are at lower altitudes in the Carpathian Mountains compared with the Alps (Ellenberg, 1988; Pawłowski, 1972).

The species distributions were also correlated with temperature seasonality, which in the studied region is also related to the precipitation pattern (Appendix S4, Table S3). *Solidago canadensis* is more abundant in the western part of the study region, which has lower temperature seasonality and higher precipitation, while *S*. *gigantea* avoids the southern part of the region with higher precipitation and also lower temperature seasonality. Previous studies examining the potential range of this species in Europe (Weber, 2001) suggested that these aspects (continentality gradient and precipitation) did not restrict their distribution in this part of Europe. Therefore, the extent to which the observed relation is causal is not clear, and the possibility exists that it reflects a peculiarity of the distribution in the studied region.

The models did not indicate that soil properties and land relief features are among the crucial factors explaining the distributions of the invaders. Both species are known to have rather broad tolerance to soils (Szymura & Szymura, 2016; Weber & Jakobs, 2005; Werner et al., 1980), which could explain why soil properties were not relevant in studied region. Observations from early phase of invasion on studied region, up to 1989s, underlined the role of river valleys, as a main route of invasion (Tokarska‐Guzik, 2005). The results obtained here show that the species are broadly widespread and their invasion is no longer related to watercourses.

#### 4.1.3 Biotic factors

Because of the character of the data (observation for 2 × 2 km grid), we had no detailed information regarding invaded habitats. However, the data still allowed testing the hypothesis regarding species co‐occurrence at landscape scale and the effect of dominant land cover/land use forms. Results from other region of Central Europe revealed existence of large areas dominated by a single invasive *Solidago* species, where the presence of another was unlikely. This spatial pattern results, most likely, from priority effect (Szymura & Szymura, 2016). In the studied region, we had no evidences for such phenomenon: The presence of one species did not explain the absence of the other. The species rarely formed mixed stands (Szymura & Szymura, 2016), but considering grain size used in this examination (square 2 × 2 km) it can be assumed that they could co‐occur in the same landscape. We also found that the presence of *S*. *canadensis* is rather unlikely in a landscape dominated by agricultural areas. It could be linked to high use of herbicides and a small amount of available area for invasive goldenrod habitats (e.g., abandoned fields, meadow, pastures) in lands with intense, large‐scale agriculture (Szymura & Szymura, 2016; Szymura, Szymura, & Wolski, 2016).

The relatively low importance of variables that can be related to biotic interactions does not necessarily mean that biotic interactions did not shape invasion pattern. It is more likely related to the grid size in this study (2 × 2 km), while the biotic interactions occur mostly in the closest vicinity of the studied individuals. Such data can potentially be derived from other sources of information, namely phytosociological relevés, which document species composition and abundance in small plots (~25 m^2^ for herbal vegetation).

### 4.2 Conservation implications

The two species differed regarding prominent constraints: both were limited climatically, avoiding cold, mountain climate, but *S*. *canadensis* with a still limited range was also related to proxies of human pressure. Based on the results, it can be hypothesized that recent dispersal of *S*. *gigantea* in the studied region has happened mostly spontaneously, while the spread of *S*. *canadensis* could be related to trade and intentional introductions. Given the wide range of distribution of both species, their successful eradication in the region seems unlikely. The eradication of *Solidago* is not easy and must include the establishment of native vegetation to prevent reinvasion of the *Solidago* on the site. It needs a long time and financial effort (Szymura et al., 2019; Szymura, Szymura, & Wolski, 2016). However, local eradication in mountains, above 600–850 m a.s.l. where the species occur infrequently may still be feasible and could be considered as a management option. In the case of *S*. *canadensis*, proscription of its sale could restrict its further spread. Assuming the successful restriction of the trade, eradication in the eastern and central parts of the region, where the species is still uncommon, will be achievable. Similarly, the control of invasive plant species populations in human settlements and their surrounding area seems to be a reasonable method. In contrast, the management of *S*. *gigantea* should focus on areas with a high value for nature conservation that are close to already existing populations of this species. Among management of invasive *Solidago* stands, the mowing, grazing, flooding, and combination of these methods are considered (Nagy et al., 2020, 2021). Herbicide use should be banned because of its environmental impact, including the effect on native vegetation (Schulz et al., 2021; Weidlich et al., 2020); moreover, its long‐term effect is not better than mechanic methods (Szymura et al., 2019). Nonetheless, the model's prediction suggests a considerable increase of invaded areas by both species. The location of suitable squares that are not yet colonized suggests that the expansion of the invaders will take place by range filing rather than increasing the range (Appendix S7, Map S4). In Central Europe especially prone to invasion are abandoned agricultural lands (Bartha et al., 2014; Fenesi et al., 2015), therefore a policy preventing agricultural land abandonment is desirable to counteract the further increase of goldenrods invasion level. The model outputs seem to be transferable into other areas with similar climate, land use history, economy, and invasion history, including the Carpathian Mountains and the surrounding regions in Slovakia, Ukraine, Hungary, and Romania. However, we did not have enough data to directly test the possible model application, using the limited number of explanatory variables, to maximize the iSDM transferability (Petitpierre et al., 2017). In this context, the procedure of model simplification (Elith et al., 2008), which reduced the number of explanatory variables, seems to be a great advantage of the BRT modeling technique.

### 4.3 Model limitations, and methodological problems

Recently, numerous iSDMs have been based on presence‐only data and employ so‐called background points (pseudo‐absences). Nonetheless, data not only on species presence but also their true (i.e., confirmed) absence are considered more relevant for modeling (Barbet‐Massin et al., 2012; Brotons et al., 2004; Elith et al., 2020). Unfortunately, confirmed absence data are problematic because they need a high sampling effort (Barbet‐Massin et al., 2012; MacKenzie & Royle, 2005) to be realistic. Our results show that exclusion of squares with low sampling effort improves the model's performance. This suggests an issue of sampling bias, which can be ameliorated by appropriate procedures. Our approach seems to be promising, but it needs further study in order to better understand its operation. The typical assumption, such as higher sampling effort in densely populated areas and near roads, is not adequate for invasive species because they typically occur in urban areas and along communication routes (Niinemets & Peñuelas, 2008; Szymura, Szymura, & Wolski, 2016).

Another problem consists of causality in our model: The approach applied represents a correlative type of model that is unable to directly capture the underlying processes driving the observed patterns of distribution. Contrary to the correlative approach, the mechanistic (or process‐based) models, which are built using explicit descriptions of biological mechanisms, are free from this disadvantage (Yates et al., 2018). In result, mechanistic and hybrid models have recently been recommended for modeling species distribution (Zurell et al., 2016). Studies in simulated systems reveal great potential of mechanistic models as BioGEEM in examination of eco‐ecological questions (Cabral et al., 2019). However, they still meet a numerous obstacles in practical implementation, especially in macroecological and biogeographical applications (Cabral et al., 2017), since simulation of large, species‐rich ecosystems is challenging (Cabral et al., 2019). They need appropriate formulation including detailed data on species response to environment, preferably coming from experiments, which are typically unavailable (Yates et al., 2018; Zurell et al., 2016). In practice, the models rely to a considerable degree on parametrization based on observational data, and as a result, the difference between correlative and mechanistic models is often fuzzy (Yates et al., 2018). Similarly, there is a problem with incorporating the effect of long‐range seed dispersal by wind. It needs detailed data regarding wind direction and velocity, seed dispersal kernel, and local population demography (Neubert & Caswell, 2000). As result, models are developed for restricted areas (e.g., Baker, 2017; Williams et al., 2008), which caused particularly useful for modeling dispersal of newly established populations (Gallien et al., 2010). To conclude, the recent state of knowledge regarding processes driving *Solidago* invasion restricts application of mechanistic or hybrid models of their invasion to a regional spatial extent.

## 5 CONCLUSIONS

The PAB framework enhanced the iSDM by helping in the selection of explanatory variables, as well as the ecological interpretation of the models. Nonetheless, in practice it needs high‐quality data that are typically unavailable to fulfill this approach, especially regarding biotic interactions. In case of plant invasion, adequate data on the biotic component could be delivered by phytosociological relevés. The employment of maps showing the historical distribution of invasive species enhanced the modeling by revealing differences in patterns of species spread into a region. In result, the model reveals that two alien species with similar ecology and biology can vary considerably in their invasion pattern due to direct human interference. Therefore, the conservation options, derived from iSDM, should be focused on a particular species, not groups of species, even if they have similar ecology and are closely related taxonomically.

The presence/absence data, in addition to their pre‐eminence compared with opportunistic, presence‐only data for species distribution modeling purposes, are still prone to some bias. Results of this study suggest that the bias is correlated with mistakenly reported species absence. Exclusion of the potentially undersampled plots increased the model performance; however, additional data are needed (e.g., richness of target species group).

## CONFLICT OF INTEREST

The authors declare no competing interest.

## AUTHOR CONTRIBUTIONS

**Peliyagodage Chathura Dineth Perera:** Conceptualization (supporting); Formal analysis (supporting); Funding acquisition (equal); Investigation (supporting); Methodology (equal); Visualization (supporting); Writing‐original draft (lead); Writing‐review & editing (lead). **Tomasz H. Szymura:** Conceptualization (equal); Data curation (lead); Formal analysis (lead); Methodology (equal); Software (equal); Supervision (equal); Visualization (equal); Writing‐original draft (equal); Writing‐review & editing (equal). **Adam Zając:** Data curation (equal); Resources (lead). **Dominika Chmolowska:** Software (equal); Visualization (equal); Writing‐review & editing (equal). **Magdalena Szymura:** Conceptualization (equal); Data curation (equal); Formal analysis (equal); Funding acquisition (equal); Investigation (equal); Supervision (lead); Visualization (equal); Writing‐original draft (equal); Writing‐review & editing (equal).

## Supporting information

Supplementary Material

## Data Availability

All data used are available publicly from the sources given in the manuscript and appendices.

## References

[ece37989-bib-0001] Bailey, J. J., Boyd, D. S., Hjort, J., Lavers, C. P., & Field, R. (2017). Modelling native and alien vascular plant species richness: At which scales is geodiversity most relevant? Global Ecology and Biogeography, 26(7), 763–776. 10.1111/geb.12574

[ece37989-bib-0002] Baker, C. M. (2017). Target the source: Optimal spatiotemporal resource allocation for invasive species control. Conservation Letters, 10(1), 41–48. 10.1111/conl.12236

[ece37989-bib-0003] Ballabio, C., Lugato, E., Fernández‐Ugalde, O., Orgiazzi, A., Jones, A., Borrelli, P., Montanarella, L., & Panagos, P. (2019). Mapping LUCAS topsoil chemical properties at European scale using Gaussian process regression. Geoderma, 355, 113912. 10.1016/j.geoderma.2019.113912 31798185 PMC6743211

[ece37989-bib-0004] Barbet‐Massin, M., Jiguet, F., Albert, C. H., & Thuiller, W. (2012). Selecting pseudo‐absences for species distribution models: How, where and how many? Methods in Ecology and Evolution, 3(2), 327–338. 10.1111/j.2041-210X.2011.00172.x

[ece37989-bib-0005] Bartha, S., Szentes, S., Horváth, A., Házi, J., Zimmermann, Z., Molnár, C., Dancza, I., Margóczi, K., Pál, R. W., Purger, D., Schmidt, D., Óvári, M., Komoly, C., Sutyinszki, Z., Szabó, G., Csathó, A. I., Juhász, M., Penksza, K., & Molnár, Z. (2014). Impact of mid‐successional dominant species on the diversity and progress of succession in regenerating temperate grasslands. Applied Vegetation Science, 17, 201–213. 10.1111/avsc.12066

[ece37989-bib-0006] Basnou, C., Iguzquiza, J., & Pino, J. (2015). Examining the role of landscape structure and dynamics in alien plant invasion from urban Mediterranean coastal habitats. Landscape and Urban Planning, 136, 156–164. 10.1016/j.landurbplan.2014.12.001

[ece37989-bib-0007] Bazzichetto, M., Malavasi, M., Barták, V., Acosta, A. T. R., Moudrý, V., & Carranza, M. L. (2018). Modeling plant invasion on Mediterranean coastal landscapes: An integrative approach using remotely sensed data. Landscape and Urban Planning, 171, 98–106. 10.1016/j.landurbplan.2017.11.006

[ece37989-bib-0008] Beaury, E. M., Finn, J. T., Corbin, J. D., Barr, V., & Bradley, B. A. (2020). Biotic resistance to invasion is ubiquitous across ecosystems of the United States. Ecology Letters, 23(3), 476–482. 10.1111/ele.13446 31875651

[ece37989-bib-0009] Bielecka, A., Królak, E., & Biardzka, E. (2017). Habitat conditions of Canadian goldenrod in a selected region of eastern Poland. Journal of Ecological Engineering, 18(4), 76–81. 10.12911/22998993/74284

[ece37989-bib-0010] Blackburn, T. M., Pyšek, P., Bacher, S., Carlton, J. T., Duncan, R. P., Jarošík, V., Wilson, J. R. U., & Richardson, D. M. (2011). A proposed unified framework for biological invasions. Trends in Ecology and Evolution, 26(7), 333–339. 10.1016/j.tree.2011.03.023 21601306

[ece37989-bib-0011] Brotons, L., Thuiller, W., Araújo, M. B., & Hirzel, A. H. (2004). Presence‐absence versus presence‐only modelling methods for predicting bird habitat suitability. Ecography, 27(4), 437–448. 10.1111/j.0906-7590.2004.03764.x

[ece37989-bib-0012] CABI . (2018). Solidago canadensis L. Retrieved from https://www.cabi.org/isc/datasheet/50599

[ece37989-bib-0013] Cabral, J. S., Valente, L., & Hartig, F. (2017). Mechanistic simulation models in macroecology and biogeography: State‐of‐art and prospects. Ecography, 40(2), 267–280. 10.1111/ecog.02480

[ece37989-bib-0014] Cabral, J. S., Wiegand, K., & Kreft, H. (2019). Interactions between ecological, evolutionary and environmental processes unveil complex dynamics of insular plant diversity. Journal of Biogeography, 46(7), 1582–1597. 10.1111/jbi.13606

[ece37989-bib-0015] Catford, J. A., Jansson, R., & Nilsson, C. (2009). Reducing redundancy in invasion ecology by integrating hypotheses into a single theoretical framework. Diversity and Distributions, 15(1), 22–40. 10.1111/j.1472-4642.2008.00521.x

[ece37989-bib-0016] Chamberlain, S. A., Bronstein, J. L., & Rudgers, J. A. (2014). How context dependent are species interactions? Ecology Letters, 17(7), 881–890. 10.1111/ele.12279 24735225

[ece37989-bib-0017] Chapman, D., Pescott, O. L., Roy, H. E., & Tanner, R. (2019). Improving species distribution models for invasive non‐native species with biologically informed pseudo‐absence selection. Journal of Biogeography, 46(5), 1029–1040. 10.1111/jbi.13555

[ece37989-bib-0018] Charles, H., & Dukes, J. S. (2007). Impacts of invasive species on ecosystem services. Biological Invasions, 193, 217–237. 10.1007/978-3-540-36920-2_13

[ece37989-bib-0019] Chmura, D., Dyba, P., Kraj, P., Peplińska, N., Pilorz, A., & Roman, M. (2016). Invasion of alien *Solidago* taxa into urban habitats: A study of selected towns in Southern Poland. Chemistry‐Didactics‐Ecology‐Metrology, 20(1–2), 97–104. 10.1515/cdem-2015-0010

[ece37989-bib-0020] Chytrý, M., Jarošík, V., Pyšek, P., Hájek, O., Knollová, I., Tichý, L., & Danihelka, J. (2008). Separating habitat invasibility by alien plants from the actual level of invasion. Ecology, 89(6), 1541–1553. 10.1890/07-0682.1 18589519

[ece37989-bib-0021] Chytrý, M., Pyšek, P., Wild, J., Pino, J., Maskell, L. C., & Vilà, M. (2009). European map of alien plant invasions based on the quantitative assessment across habitats. Diversity and Distributions, 15(1), 98–107. 10.1111/j.1472-4642.2008.00515.x

[ece37989-bib-0022] Czarniecka‐Wiera, M., Szymura, T. H., & Kącki, Z. (2020). Understanding the importance of spatial scale in the patterns of grassland invasions. Science of the Total Environment, 727, 138669. 10.1016/j.scitotenv.2020.138669 32325319

[ece37989-bib-0023] Davis, M. A., Grime, J. P., & Thompson, K. (2000). Fluctuating resources in plant communities: A general theory of invasibility. Journal of Ecology, 88(3), 528–534. 10.1046/j.1365-2745.2000.00473.x

[ece37989-bib-0024] de Groot, M., Kleijn, D., & Jogan, N. (2007). Species groups occupying different trophic levels respond differently to the invasion of semi‐natural vegetation by *Solidago canadensis* . Biological Conservation, 136(4), 612–617. 10.1016/j.biocon.2007.01.005

[ece37989-bib-0025] De’Ath, G. (2007). Boosted trees for ecological modeling and prediction. Ecology, 88(1), 243–251. 17489472 10.1890/0012-9658(2007)88[243:btfema]2.0.co;2

[ece37989-bib-0026] De’Ath, G., & Fabricius, K. E. (2000). Classification and regression trees: A powerful yet simple technique for ecological data analysis. Ecology, 81(11), 3178–3192.

[ece37989-bib-0027] Dormann, C. F., Elith, J., Bacher, S., Buchmann, C., Carl, G., Carré, G., Marquéz, J. R. G., Gruber, B., Lafourcade, B., Leitão, P. J., Münkemüller, T., McClean, C., Osborne, P. E., Reineking, B., Schröder, B., Skidmore, A. K., Zurell, D., & Lautenbach, S. (2013). Collinearity: A review of methods to deal with it and a simulation study evaluating their performance. Ecography, 36(1), 27–46. 10.1111/j.1600-0587.2012.07348.x

[ece37989-bib-0028] Elith, J., Ferrier, S., Huettmann, F., & Leathwick, J. (2005). The evaluation strip: A new and robust method for plotting predicted responses from species distribution models. Ecological Modelling, 186(3), 280–289. 10.1016/j.ecolmodel.2004.12.007

[ece37989-bib-0029] Elith, J., Graham, C., Valavi, R., Abegg, M., Bruce, C., Ford, A., Guisan, A., Hijmans, R. J., Huettmann, F., Lohmann, L., Loiselle, B., Moritz, C., Overton, J., Peterson, A. T., Phillips, S., Richardson, K., Williams, S., Wiser, S. K., Wohlgemuth, T., & Zimmermann, N. E. (2020). Presence‐only and presence‐absence data for comparing species distribution modeling methods. Biodiversity Informatics, 15(2), 69–80. 10.17161/bi.v15i2.13384

[ece37989-bib-0030] Elith, J., Leathwick, J. R., & Hastie, T. (2008). A working guide to boosted regression trees. Journal of Animal Ecology, 77(4), 802–813. 10.1111/j.1365-2656.2008.01390.x 18397250

[ece37989-bib-0031] Ellenberg, H. H. (1988). Vegetation ecology of central Europe. Cambridge University Press.

[ece37989-bib-0032] EPPO . (2020). European and Mediterranean plant protection organisation invasive species alert list. Retrieved from https://www.eppo.int/ACTIVITIES/plant_quarantine/alert_list

[ece37989-bib-0033] Essl, F., Dullinger, S., Rabitsch, W., Hulme, P. E., Hülber, K., Jarošík, V., Kleinbauer, I., Krausmann, F., Kühn, I., Nentwig, W., Vilà, M., Genovesi, P., Gherardi, F., Desprez‐Loustau, M.‐L., Roques, A., & Pyšek, P. (2011). Socioeconomic legacy yields an invasion debt. Proceedings of the National Academy of Sciences of the United States of America, 108(1), 203–207. 10.1073/pnas.1011728108 21173227 PMC3017203

[ece37989-bib-0034] Fenesi, A., Vágási, C. I., Beldean, M., Földesi, R., Kolcsár, L. P., Shapiro, J. T., Török, E., & Kovács‐Hostyánszki, A. (2015). *Solidago canadensis* impacts on native plant and pollinator communities in different‐aged old fields. Basic and Applied Ecology, 16(4), 335–346. 10.1016/j.baae.2015.03.003

[ece37989-bib-0035] Foxcroft, L. C., Pickett, S. T. A., & Cadenasso, M. L. (2011). Expanding the conceptual frameworks of plant invasion ecology. Perspectives in Plant Ecology, Evolution and Systematics, 13(2), 89–100. 10.1016/j.ppees.2011.03.004

[ece37989-bib-0036] Frost, C. M., Allen, W. J., Courchamp, F., Jeschke, J. M., Saul, W. C., & Wardle, D. A. (2019). Using network theory to understand and predict biological invasions. Trends in Ecology & Evolution, 34(9), 831–843. 10.1016/j.tree.2019.04.012 31155422

[ece37989-bib-0037] Funk, J. L., Cleland, E. E., Suding, K. N., & Zavaleta, E. S. (2008). Restoration through reassembly: Plant traits and invasion resistance. Trends in Ecology and Evolution, 23, 695–703. 10.1016/j.tree.2008.07.013 18951652

[ece37989-bib-0038] Gallien, L., Douzet, R., Pratte, S., Zimmermann, N. E., & Thuiller, W. (2012). Invasive species distribution models–how violating the equilibrium assumption can create new insights. Global Ecology and Biogeography, 21(11), 1126–1136. 10.1111/j.1466-8238.2012.00768.x

[ece37989-bib-0039] Gallien, L., Münkemüller, T., Albert, C. H., Boulangeat, I., & Thuiller, W. (2010). Predicting potential distributions of invasive species: Where to go from here? Diversity and Distributions, 16(3), 331–342. 10.1111/j.1472-4642.2010.00652.x

[ece37989-bib-0040] González‐Moreno, P., Pino, J., Carreras, D., Basnou, C., Fernández‐Rebollar, I., & Vilà, M. (2013). Quantifying the landscape influence on plant invasions in Mediterranean coastal habitats. Landscape Ecology, 28(5), 891–903. 10.1007/s10980-013-9857-1

[ece37989-bib-0041] Greenwell, B., Boehmke, B., Cunningham, J., & GBM Developers . (2020). gbm: Generalized boosted regression models. R package version 2.1.8. Retrieved from https://CRAN.R‐project.org/package=gbm

[ece37989-bib-0042] Guisan, A., Zimmermann, N. E., Elith, J., Graham, C. H., Phillips, S., & Peterson, A. T. (2007). What matters for predicting the occurrences of trees: Techniques, data, or species' characteristics? Ecological Monographs, 77(4), 615–630. 10.1890/06-1060.1

[ece37989-bib-0043] Gusev, A. P. (2015). The impact of invasive Canadian goldenrod (*Solidago canadensis* L.) on regenerative succession in old fields (the Southeast of Belarus). Russian Journal of Biological Invasions, 6(2), 74–77. 10.1134/S2075111715020034

[ece37989-bib-0044] Guzikowa, M., & Maycock, P. F. (1986). The invasion and expansion of three North American species of goldenrod (*Solidago canadensis* L. sensu lato. *S. gigantea* Ait. and *S. graminifolia* (L) Salisb) in Poland. Acta Societatis Botanicorum Poloniae, 55(3), 367–384. 10.5586/asbp.1986.034

[ece37989-bib-0045] Hattab, T., Garzón‐López, C. X., Ewald, M., Skowronek, S., Aerts, R., Horen, H., Brasseur, B., Gallet‐Moron, E., Spicher, F., Decocq, G., Feilhauer, H., Honnay, O., Kempeneers, P., Schmidtlein, S., Somers, B., Van De Kerchove, R., Rocchini, D., & Lenoir, J. (2017). A unified framework to model the potential and realized distributions of invasive species within the invaded range. Diversity and Distributions, 23(7), 806–819. 10.1111/ddi.12566

[ece37989-bib-0046] Hejda, M., & de Bello, F. (2013). Impact of plant invasions on functional diversity in the vegetation of Central Europe. Journal of Vegetation Science, 24(5), 890–897. 10.1111/jvs.12026

[ece37989-bib-0047] Hejda, M., Pyšek, P., & Jarošík, V. (2009). Impact of invasive plants on the species richness, diversity and composition of invaded communities. Journal of Ecology, 97(3), 393–403. 10.1111/j.1365-2745.2009.01480.x

[ece37989-bib-0048] Hijmans, R. J., Cameron, S. E., Parra, J. L., Jones, P. G., & Jarvis, A. (2005). Very high resolution interpolated climate surfaces for global land areas. International Journal of Climatology: A Journal of the Royal Meteorological Society, 25(15), 1965–1978. 10.1002/joc.1276

[ece37989-bib-0049] Hijmans, R. J., Phillips, S., Leathwick, J., & Elith, J. (2020). dismo: Species distribution modeling. R package version 1.3‐3. Retrieved from https://CRAN.R‐project.org/package=dismo

[ece37989-bib-0050] Hulme, P. E. (2009). Trade, transport and trouble: Managing invasive species pathways in an era of globalization. Journal of Applied Ecology, 46(1), 10–18. 10.1111/j.1365-2664.2008.01600.x

[ece37989-bib-0051] Hulme, P. E. (2017). Climate change and biological invasions: Evidence, expectations, and response options. Biological Reviews, 3, 1297–1313. 10.1111/brv.12282 27241717

[ece37989-bib-0052] Johnson, M. F. (1995). Goldenrods in Virginia: *Euthamia* (Nutt.) Nutt. and *Solidago* L. Castanea, 60(2), 114–140.

[ece37989-bib-0053] Kajzer‐Bonk, J., Szpiłyk, D., & Woyciechowski, M. (2016). Invasive goldenrods affect abundance and diversity of grassland ant communities (Hymenoptera: Formicidae). Journal of Insect Conservation, 20(1), 99–105. 10.1007/s10841-016-9843-4

[ece37989-bib-0054] Kuebbing, S. E., & Nuñez, M. A. (2015). Negative, neutral, and positive interactions among nonnative plants: Patterns, processes, and management implications. Global Change Biology, 21(2), 926–934. 10.1111/gcb.12711 25142018

[ece37989-bib-0055] Kulmatiski, A., Beard, K. H., & Stark, J. M. (2006). Soil history as a primary control on plant invasion in abandoned agricultural fields. Journal of Applied Ecology, 43(5), 868–876. 10.1111/j.1365-2664.2006.01192.x

[ece37989-bib-0056] Le Maitre, D. C., Richardson, D. M., & Chapman, R. A. (2004). Alien plant invasions in South Africa: Driving forces and the human dimension: Working for water. South African Journal of Science, 100(1–2), 103–112.

[ece37989-bib-0057] Ledger, K. J., Pal, R. W., Murphy, P., Nagy, D. U., Filep, R., & Callaway, R. M. (2015). Impact of an invader on species diversity is stronger in the non‐native range than in the native range. Plant Ecology, 216(9), 1285–1295. 10.1007/s11258-015-0508-2

[ece37989-bib-0058] Lenda, M., Skórka, P., Knops, J. M., Moroń, D., Sutherland, W. J., Kuszewska, K., & Woyciechowski, M. (2014). Effect of the internet commerce on dispersal modes of invasive alien species. PLoS One, 9(6), e99786. 10.1371/journal.pone.0099786 24932498 PMC4059692

[ece37989-bib-0059] Lenda, M., Skórka, P., Kuszewska, K., Moroń, D., Bełcik, M., Baczek Kwinta, R., Janowiak, F., Duncan, D. H., Vesk, P. A., Possingham, H. P., & Knops, J. M. (2020). Misinformation, internet honey trading and beekeepers drive a plant invasion. Ecology Letters, 24(2), 165–169. 10.1111/ele.13645 33201583

[ece37989-bib-0060] Lenda, M., Witek, M., Skórka, P., Moroń, D., & Woyciechowski, M. (2013). Invasive alien plants affect grassland ant communities, colony size and foraging behaviour. Biological Invasions, 15(11), 2403–2414. 10.1007/s10530-013-0461-8

[ece37989-bib-0061] Linders, T. E. W., Schaffner, U., Eschen, R., Abebe, A., Choge, S. K., Nigatu, L., Mbaabu, P. R., Shiferaw, H., & Allan, E. (2019). Direct and indirect effects of invasive species: Biodiversity loss is a major mechanism by which an invasive tree affects ecosystem functioning. Journal of Ecology, 107(6), 2660–2672. 10.1111/1365-2745.13268

[ece37989-bib-0062] Lozano, V., Marzialetti, F., Carranza, M. L., Chapman, D., Branquart, E., Dološ, K., Große‐Stoltenberg, A., Fiori, M., Capece, P., & Brundu, G. (2020). Modelling *Acacia saligna* invasion in a large Mediterranean island using PAB factors: A tool for implementing the European legislation on invasive species. Ecological Indicators, 116, 106516. 10.1016/j.ecolind.2020.106516

[ece37989-bib-0063] MacArthur, R., & Levins, R. (1967). The limiting similarity, convergence, and divergence of coexisting species. The American Naturalist, 101(921), 377–385. 10.2307/2459090

[ece37989-bib-0064] MacKenzie, D. I., & Royle, J. A. (2005). Designing occupancy studies: General advice and allocating survey effort. Journal of Applied Ecology, 42(6), 1105–1114. 10.1111/j.1365-2664.2005.01098.x

[ece37989-bib-0065] McKinney, M. L. (2008). Effects of urbanization on species richness: A review of plants and animals. Urban Ecosystems, 11(2), 161–176. 10.1007/s11252-007-0045-4

[ece37989-bib-0066] Meyer, A. H., & Schmid, B. (1999a). Seed dynamics and seedling establishment in the invading perennial *Solidago altissima* under different experimental treatments. Journal of Ecology, 87(1), 28–41. 10.1046/j.1365-2745.1999.00316.x

[ece37989-bib-0067] Meyer, A. H., & Schmid, B. (1999b). Experimental demography of the old‐field perennial *Solidago altissima*: The dynamics of the shoot population. Journal of Ecology, 87(1), 17–27. 10.1046/j.1365-2745.1999.00315.x

[ece37989-bib-0068] Milbau, A., Stout, J. C., Graae, B. J., & Nijs, I. (2009). A hierarchical framework for integrating invasibility experiments incorporating different factors and spatial scales. Biological Invasions, 11(4), 941–950. 10.1007/s10530-008-9306-2

[ece37989-bib-0069] Moran, E. V., Reid, A., & Levine, J. M. (2017). Population genetics and adaptation to climate along elevation gradients in invasive *Solidago canadensis* . PLoS One, 12(9), e0185539. 10.1371/journal.pone.0185539 28957402 PMC5619793

[ece37989-bib-0070] Moroń, D., Lenda, M., Skórka, P., Szentgyörgyi, H., Settele, J., & Woyciechowski, M. (2009). Wild pollinator communities are negatively affected by invasion of alien goldenrods in grassland landscapes. Biological Conservation, 142(7), 1322–1332. 10.1016/j.biocon.2008.12.036

[ece37989-bib-0071] Moroń, D., Marjańska, E., Skórka, P., Lenda, M., & Woyciechowski, M. (2021). Invader–pollinator paradox: Invasive goldenrods benefit from large size pollinators. Diversity and Distributions, 27(4), 632–641. 10.1111/ddi.13221

[ece37989-bib-0072] Nagy, D. U., Rauschert, E. S., Callaway, R. M., Henn, T., Filep, R., & Pal, R. W. (2021). Intense mowing management suppresses invader, but shifts competitive resistance by a native to facilitation. Restoration Ecology, e13483. 10.1111/rec.13483

[ece37989-bib-0073] Nagy, D. U., Rauschert, E. S., Henn, T., Cianfaglione, K., Stranczinger, S., & Pal, R. W. (2020). The more we do, the less we gain? Balancing effort and efficacy in managing the *Solidago gigantea* invasion. Weed Research, 60(3), 232–240. 10.1111/wre.12417

[ece37989-bib-0074] Neubert, M. G., & Caswell, H. (2000). Demography and dispersal: Calculation and sensitivity analysis of invasion speed for structured populations. Ecology, 81(6), 1613–1628.

[ece37989-bib-0075] Niinemets, Ü., & Peñuelas, J. (2008). Gardening and urban landscaping: Significant players in global change. Trends in Plant Science, 13(2), 60–65. 10.1016/j.tplants.2007.11.009 18262823

[ece37989-bib-0076] Ööpik, M., Bunce, R. G. B., & Tischler, M. (2013). Horticultural markets promote alien species invasions: An Estonian case study of herbaceous perennials. NeoBiota, 17, 19. 10.3897/neobiota.17.4217

[ece37989-bib-0077] Pawłowski, B. (1972). Szata roślinna gór polskich. In W. Szafer, & K. Zarzycki (Eds.), Szata roślinna Polski (pp. 189–253). PWN.

[ece37989-bib-0078] Pejchar, L., & Mooney, H. A. (2009). Invasive species, ecosystem services and human well‐being. Trends in Ecology & Evolution, 24(9), 497–504. 10.1016/j.tree.2009.03.016 19577817

[ece37989-bib-0079] Peltzer, D. A., Kurokawa, H., & Wardle, D. A. (2016). Soil fertility and disturbance interact to drive contrasting responses of co‐occurring native and nonnative species. Ecology, 97(2), 515–529. 10.1890/15-0298.1 27145625

[ece37989-bib-0080] Perkins, L. B., Leger, E. A., & Nowak, R. S. (2011). Invasion triangle: An organizational framework for species invasion. Ecology and Evolution, 1(4), 610–625. 10.1002/ece3.47 22393528 PMC3287335

[ece37989-bib-0081] Petitpierre, B., Broennimann, O., Kueffer, C., Daehler, C., & Guisan, A. (2017). Selecting predictors to maximize the transferability of species distribution models: Lessons from cross‐continental plant invasions. Global Ecology and Biogeography, 26(3), 275–287. 10.1111/geb.12530

[ece37989-bib-0082] Phillips, S. J., Dudík, M., Elith, J., Graham, C. H., Lehmann, A., Leathwick, J., & Ferrier, S. (2009). Sample selection bias and presence‐only distribution models: Implications for background and pseudo‐absence data. Ecological Applications, 19(1), 181–197. 10.1890/07-2153.1 19323182

[ece37989-bib-0083] Pino, J., Font, X., Carbo, J., Jové, M., & Pallares, L. (2005). Large‐scale correlates of alien plant invasion in Catalonia (NE of Spain). Biological Conservation, 122(2), 339–350. 10.1016/j.biocon.2004.08.006

[ece37989-bib-0084] Pollnac, F., Seipel, T., Repath, C., & Rew, L. J. (2012). Plant invasion at landscape and local scales along roadways in the mountainous region of the Greater Yellowstone Ecosystem. Biological Invasions, 14(8), 1753–1763. 10.1007/s10530-012-0188-y

[ece37989-bib-0085] Pysek, P., Jarosik, V., Hulme, P. E., Kuhn, I., Wild, J., Arianoutsou, M., Bacher, S., Chiron, F., Didziulis, V., Essl, F., Genovesi, P., Gherardi, F., Hejda, M., Kark, S., Lambdon, P. W., Desprez‐Loustau, M.‐L., Nentwig, W., Pergl, J., Poboljsaj, K., … Winter, M. (2010). Disentangling the role of environmental and human pressures on biological invasions across Europe. Proceedings of the National Academy of Sciences of the United States of America, 107(27), 12157–12162. 10.1073/pnas.1002314107 20534543 PMC2901442

[ece37989-bib-0086] Pyšek, P., & Richardson, D. M. (2010). Invasive species, environmental change and management, and health. Annual Review of Environment and Resources, 35, 25–55. 10.1146/annurev-environ-033009-095548

[ece37989-bib-0087] Regos, A., Gagne, L., Alcaraz‐Segura, D., Honrado, J. P., & Domínguez, J. (2019). Effects of species traits and environmental predictors on performance and transferability of ecological niche models. Scientific Reports, 9(1), 1–14. 10.1038/s41598-019-40766-5 30862919 PMC6414724

[ece37989-bib-0088] Rejmánek, M. (1989). Invasibility of plant communities. In J. A. Drake, F. Di Castri, R. H. Groves, F. J. Kruger, H. A. Mooney, M. Rejmanek, & M. H. Williamson (Eds.), Ecology of biological invasion: A global perspective (pp. 369–388). Wiley and Sons.

[ece37989-bib-0089] Roberts, D. R., Bahn, V., Ciuti, S., Boyce, M. S., Elith, J., Guillera‐Arroita, G., Hauenstein, S., Lahoz‐Monfort, J. J., Schröder, B., Thuiller, W., Warton, D. I., Wintle, B. A., Hartig, F., & Dormann, C. F. (2017). Cross‐validation strategies for data with temporal, spatial, hierarchical, or phylogenetic structure. Ecography, 40(8), 913–929. 10.1111/ecog.02881

[ece37989-bib-0090] Roháčová, M., & Drozd, P. (2009). How many heteropteran species can live on alien goldenrods *Solidago canadensis* and *S. gigantea* in Europe? Biologia, 64(5), 981–993. 10.2478/s11756-009-0151-2

[ece37989-bib-0091] Sala, O. E., Chapin, F. S., Armesto, J. J., Berlow, E., Bloomfield, J., Dirzo, R., Huber‐Sanwald, E., Huenneke, L. F., Jackson, R. B., Kinzig, A., & Leemans, R. (2000). Global biodiversity scenarios for the year 2100. Science, 287(5459), 1770–1774. 10.1126/science.287.5459.1770 10710299

[ece37989-bib-0092] Schmid, B., Puttick, G. M., Burgess, K. H., & Bazzaz, F. A. (1988). Correlations between genet architecture and some life history features in three species of *Solidago* . Oecologia, 75(3), 459–464. 10.1007/BF00376952 28312697

[ece37989-bib-0093] Schulz, R., Bub, S., Petschick, L. L., Stehle, S., & Wolfram, J. (2021). Applied pesticide toxicity shifts toward plants and invertebrates, even in GM crops. Science, 372(6537), 81–84. 10.1126/science.abe1148 33795455

[ece37989-bib-0094] Seebens, H., Essl, F., Dawson, W., Fuentes, N., Moser, D., Pergl, J., Pyšek, P., van Kleunen, M., Weber, E., Winter, M., & Blasius, B. (2015). Global trade will accelerate plant invasions in emerging economies under climate change. Global Change Biology, 21(11), 4128–4140. 10.1111/gcb.13021 26152518

[ece37989-bib-0095] Sheppard, A. W., Shaw, R. H., & Sforza, R. (2006). Top 20 environmental weeds for classical biological control in Europe: A review of opportunities, regulations and other barriers to adoption. Weed Research, 46(2), 93–117. 10.1111/j.1365-3180.2006.00497.x

[ece37989-bib-0096] Shiferaw, H., Schaffner, U., Bewket, W., Alamirew, T., Zeleke, G., Teketay, D., & Eckert, S. (2019). Modelling the current fractional cover of an invasive alien plant and drivers of its invasion in a dryland ecosystem. Scientific Reports, 9(1), 1–12. 10.1038/s41598-018-36587-7 30733452 PMC6367408

[ece37989-bib-0097] Šimundić, A. M. (2009). Measures of diagnostic accuracy: Basic definitions. EJIFCC, 19(4), 203. 27683318 PMC4975285

[ece37989-bib-0098] Skórka, P., Lenda, M., & Tryjanowski, P. (2010). Invasive alien goldenrods negatively affect grassland bird communities in Eastern Europe. Biological Conservation, 143(4), 856–861. 10.1016/j.biocon.2009.12.030

[ece37989-bib-0099] Štajerová, K., Šmilauer, P., Brůna, J., & Pyšek, P. (2017). Distribution of invasive plants in urban environment is strongly spatially structured. Landscape Ecology, 32(3), 681–692. 10.1007/s10980-016-0480-9

[ece37989-bib-0100] Szabo‐Takacs, B., Farda, A., Zahradníček, P., & Štěpánek, P. (2015). Continentality in Europe according to various resolution regional climate models with A1B scenario in the 21st century. Quarterly Journal of the Hungarian Meteorological Service, 119(4), 515–535.

[ece37989-bib-0101] Szymura, M., Świerszcz, S., Szymura, T. H., & Jarczak, D. (2019). How to establish grassland on sites invaded by Solidago: results of a five‐year experiment. In P. Pyšek, J. Pergl, & D. Moodley (Eds.), 15th Ecology and Management of Alien Plant invasions (EMAPi) book of abstracts: Integrating research, management and policy (p. 82). Institute of Botany, Czech Academy of Sciences.

[ece37989-bib-0102] Szymura, M., & Szymura, T. H. (2013). Soil preferences and morphological diversity of goldenrods (*Solidago* L.) from south‐western Poland. Acta Societatis Botanicorum Poloniae, 82(2), 107–115. 10.5586/asbp.2013.005

[ece37989-bib-0103] Szymura, M., & Szymura, T. H. (2016). Historical contingency and spatial processes rather than ecological niche differentiation explain the distribution of invasive goldenrods (*Solidago* and *Euthamia*). Plant Ecology, 217(5), 565–582. 10.1007/s11258-016-0601-1

[ece37989-bib-0104] Szymura, M., Szymura, T. H., & Świerszcz, S. (2016). Do landscape structure and socio‐economic variables explain the *Solidago* invasion? Folia Geobotanica, 51(1), 13–25. 10.1007/s12224-016-9241-4

[ece37989-bib-0105] Szymura, M., Szymura, T. H., & Wolski, K. (2016). Invasive *Solidago* species: How large area do they occupy and what would be the cost of their removal? Polish Journal of Ecology, 64(1), 25–34. 10.3161/15052249PJE2016.64.1.003

[ece37989-bib-0106] Szymura, T. H., Szymura, M., Zając, M., & Zając, A. (2018). Effect of anthropogenic factors, landscape structure, land relief, soil and climate on risk of alien plant invasion at regional scale. Science of the Total Environment, 626, 1373–1381. 10.1016/j.scitotenv.2018.01.131 29898544

[ece37989-bib-0107] Taylor, K. T., Maxwell, B. D., Pauchard, A., Nuñez, M. A., Peltzer, D. A., Terwei, A., & Rew, L. J. (2016). Drivers of plant invasion vary globally: Evidence from pine invasions within six ecoregions. Global Ecology and Biogeography, 25(1), 96–106. 10.1111/geb.12391

[ece37989-bib-0108] Thuiller, W., Georges, D., Engler, R., & Lafourcade, B. (2012). Biomod: Tutorial. Retrieved from http://www.will.chez‐alice.fr/pdf/BiomodTutorial.pdf

[ece37989-bib-0109] Thuiller, W., Georges, D., Gueguen, M., Engler, R., & Breiner, F. (2020). biomod2: Ensemble platform for species distribution modeling. R package version 3.4.13.

[ece37989-bib-0110] Thuiller, W., Richardson, D. M., & Midgley, G. F. (2007). Will climate change promote alien plant invasions? Biological Invasions, 193, 197–211. 10.1007/978-3-540-36920-2_12

[ece37989-bib-0111] Tokarska‐Guzik, B. (2005). The establishment and spread of alien plant species (kenophytes) in the flora of Poland. Wydawnictwo Uniwersytetu Śląskiego.

[ece37989-bib-0112] Tokarska‐Guzik, B., Bzdęga, K., Nowak, T., Urbisz, A., Węgrzynek, B., & Dajdok, Z. (2015). Propozycja listy roślin gatunków obcych, które mogą stanowić zagrożenie dla przyrody Polski i Unii Europejskiej − Uniwersytet Śląski w Katowicach, Katowice. (The proposal lists of plants alien species that may endanger to Polish and European Union nature).

[ece37989-bib-0113] Valavi, R., Elith, J., Lahoz‐Monfort, J. J., & Guillera‐Arroita, G. (2019). Block CV: An r package for generating spatially or environmentally separated folds for k‐fold cross‐validation of species distribution models. Methods in Ecology and Evolution, 10(2), 225–232. 10.1111/2041-210X.13107

[ece37989-bib-0114] Vannette, R. L., & Fukami, T. (2014). Historical contingency in species interactions: Towards niche‐based predictions. Ecology Letters, 17(1), 115–124. 10.1111/ele.12204 24341984 PMC4344821

[ece37989-bib-0115] Vilà, M., & Ibáñez, I. (2011). Plant invasions in the landscape. Landscape Ecology, 26(4), 461–472. 10.1007/s10980-011-9585-3

[ece37989-bib-0116] Weber, E. (1997). Morphological variation of the introduced perennial *Solidago canadensis* L. sensulato (Asteraceae) in Europe. Botanical Journal of Linnean Society, 123(3), 197–210. 10.1111/j.1095-8339.1997.tb01413.x

[ece37989-bib-0117] Weber, E. (2000). Biological flora of Central Europe: *Solidago altissima* L. Flora., 195(2), 123–134. 10.1016/S0367-2530(17)30960-X

[ece37989-bib-0118] Weber, E. (2001). Current and potential ranges of three exotic goldenrods (*Solidago*) in Europe. Conservation Biology, 15(1), 122–128. 10.1111/j.1523-1739.2001.99424.x

[ece37989-bib-0119] Weber, E., & Jakobs, G. (2005). Biological flora of central Europe: *Solidago gigantea* Aiton. Flora‐Morphology, Distribution, Functional Ecology of Plants, 200(2), 109–118. 10.1016/j.flora.2004.09.001

[ece37989-bib-0120] Weidlich, E. W., Flórido, F. G., Sorrini, T. B., & Brancalion, P. H. (2020). Controlling invasive plant species in ecological restoration: A global review. Journal of Applied Ecology, 57(9), 1806–1817. 10.1111/1365-2664.13656

[ece37989-bib-0121] Werner, P. A., Bradburyt, I. A. N. K., & Grossi, R. S. (1980). The biology of Canadian weeds. 45 *Solidago canudensis* L. Canadian Journal of Plant Science, 60, 1393–1409. 10.4141/cjps80-194

[ece37989-bib-0122] Williams, N. S., Hahs, A. K., & Morgan, J. W. (2008). A dispersal‐constrained habitat suitability model for predicting invasion of alpine vegetation. Ecological Applications, 18(2), 347–359. 10.1890/07-0868.1 18488601

[ece37989-bib-0123] Woodford, D. J., Richardson, D. M., MacIsaac, H. J., Mandrak, N. E., Van Wilgen, B. W., Wilson, J. R., & Weyl, O. L. (2016). Confronting the wicked problem of managing biological invasions. NeoBiota, 31, 63. 10.3897/neobiota.31.10038

[ece37989-bib-0124] Yang, W., Ma, K., & Kreft, H. (2013). Geographical sampling bias in a large distributional database and its effects on species richness–environment models. Journal of Biogeography, 40(8), 1415–1426. 10.1111/jbi.12108

[ece37989-bib-0125] Yates, K. L., Bouchet, P. J., Caley, M. J., Mengersen, K., Randin, C. F., Parnell, S., Fielding, A. H., Bamford, A. J., Ban, S., Barbosa, A. M., Dormann, C. F., Elith, J., Embling, C. B., Ervin, G. N., Fisher, R., Gould, S., Graf, R. F., Gregr, E. J., Halpin, P. N., … Sequeira, A. M. M. (2018). Outstanding challenges in the transferability of ecological models. Trends in Ecology & Evolution, 33(10), 790–802. 10.1016/j.tree.2018.08.001 30166069

[ece37989-bib-0126] Ye, X. Q., Yan, Y. N., Wu, M., & Yu, F. H. (2019). High capacity of nutrient accumulation by invasive *Solidago canadensis* in a coastal grassland. Frontiers in Plant Science, 10, 575. 10.3389/fpls.2019.00575 31134115 PMC6514223

[ece37989-bib-0127] Zając, A., & Zając, M. (2015). Distribution of Kenophytes in the Polish Carpathians and their Foreland (p. 304). Instytut Botaniki Uniwersytetu Jagiellońskiego.

[ece37989-bib-0128] Zhang, F., & Wan, F. (2017). Canada Goldenrod *Solidago canadensis* L. In F. Wan, M. Jiang, & A. Zhan (Eds.), Biological Invasions and Its Management in China. Invading Nature ‐ Springer Series in Invasion Ecology, 13 143–151. Singapore: Springer. 10.1007/978-981-10-3427-5_10

[ece37989-bib-0129] Zihare, L., & Blumberga, D. (2017). Insight into bioeconomy. *Solidago canadensis* as a valid resource. Energy Procedia, 128, 275–280. 10.1016/j.egypro.2017.09.074

[ece37989-bib-0130] Zurell, D., Franklin, J., König, C., Bouchet, P. J., Dormann, C. F., Elith, J., Fandos, G., Feng, X., Guillera‐Arroita, G., Guisan, A., Lahoz‐Monfort, J. J., Leitão, P. J., Park, D. S., Peterson, A. T., Rapacciuolo, G., Schmatz, D. R., Schröder, B., Serra‐Diaz, J. M., Thuiller, W., … Merow, C. (2020). A standard protocol for reporting species distribution models. Ecography, 43(9), 1261–1277. 10.1111/ecog.04960

[ece37989-bib-0131] Zurell, D., Thuiller, W., Pagel, J., Cabral, J. S., Münkemüller, T., Gravel, D., Dullinger, S., Normand, S., Schiffers, K. H., Moore, K. A., & Zimmermann, N. E. (2016). Benchmarking novel approaches for modelling species range dynamics. Global Change Biology, 22(8), 2651–2664. 10.1111/gcb.13251 26872305 PMC4972146

