# Supplementary material for: Drivers of Solidago species invasion in Central Europe—Case study in the landscape of the Carpathian Mountains and their foreground
Source: Ecol Evol. 2021 Aug 10;11(18):12429–44. doi: 10.1002/ece3.7989 (PMC8462131; doi:10.1002/ece3.7989)
Supplement: Supplementary file 1 — Supplementary Material [file ECE3-11-12429-s001.docx]

**Appendix for:**

1. Invasive *Solidago* distribution data source and method for sampling effort correction

The data on invasive *Solidago* distribution were derived from atlas titled: ‘Distribution of Kenophytes in the Polish Carpathians and their Foreland’ (Zając & Zając, 2015 eds.). The ‘Distribution of Kenophytes ….’ shows maps of the distribution of 195 neophytes (alien plant species introduced after 1500 A.D.) in a 2x2 km grid in the Polish part of the Carpathian Massif and its foreland, Central Europe (the total study area is 31,920 km^2^). The total record count of 82,164 was amassed over 150 years but about 90% of the data were gathered after 1990. The fieldworks designed for the purpose of compiling the ‘Distribution of Kenophytes atlas’ were focused on a survey of flora in particular regions (e.g. mountain ranges, particular towns and surrounds). These observations were accomplished by additional data from phytosociological relevés, collected during exploration of the entire study area. The fieldwork was carried out by several dozen professional botanists as well as PhD and MSc students. During the fieldwork, the workers used a predefined 2x2 km grid for sampling. The material obtained is a sum of all these works, supervised by Zając A. and Zając M. Therefore, they represents ‘survey’ type observation, with true absence data, not ‘collection’, type according Elith et al. (2020) nomenclature. Nonetheless, there is always problem with confirmed absence data, which need a high sampling effort (Barbet‐Massin, Jiguet, Albert, & Thuiller, 2012; MacKenzie & Royle, 2005).

To reducing the potential effect of the lower recording effort in some regions on the invasive *Solidago* distribution models, we employ a ‘target group approach’ (Ponder et al., 2001). The target group consists 173 terrestrial neophytes, which distribution is presented in the ‘Distribution of Kenophytes ….’ atlas (the same atlas, which deliver data for this study). The spatial pattern of variability in neophytes richness in 2 x 2 km squares was already modelled and explained basing on environmental data (Szymura et al., 2018). We assume that the highest negative values of the model residuals from Szymura et al. (2018) study could indicates under-sampled squares (Map S.1). Since the model focused on species presence/absence in square grid, but not richness or abundance, we did not consider the highest values of the residuals which could indicate over-sampled squares. Next, we have tested the effect of exclusion of squares with the lowest values of residuals and, simultaneously, without any *Solidago* species presence, on evaluations of models explaining *S*. *canadensis* and *S*. *gigantea* distribution. For this purpose we applied boosted regression trees (BRT), with the same set of environmental data which were used in models in the article (Table 1 in the article, and Table S1 for the variables details), as explanatory variables. Basing on area under curve (AUC) values we quantified the effect of exclusion of 5, 10, 15, 20, 25 and 30% of squares on the models evaluations. Results of these preliminary modelling suggested that the lowest sum of loss of explanatory power of the models, comparing to the best models for particular species, gives the ban of 25 % of squares (that is1950 squares, Fig. S1). The BRT models for the preliminary explorations were set: tree complexity = 5, learning rate = 0.001, bag fraction = 0.5, tolerance = 0.005 and maximal number of trees 4000.

Map S1. The spatial distribution of the residuals of neophytes distribution model (Szymura et al., 2018). The four groups represent quantiles.


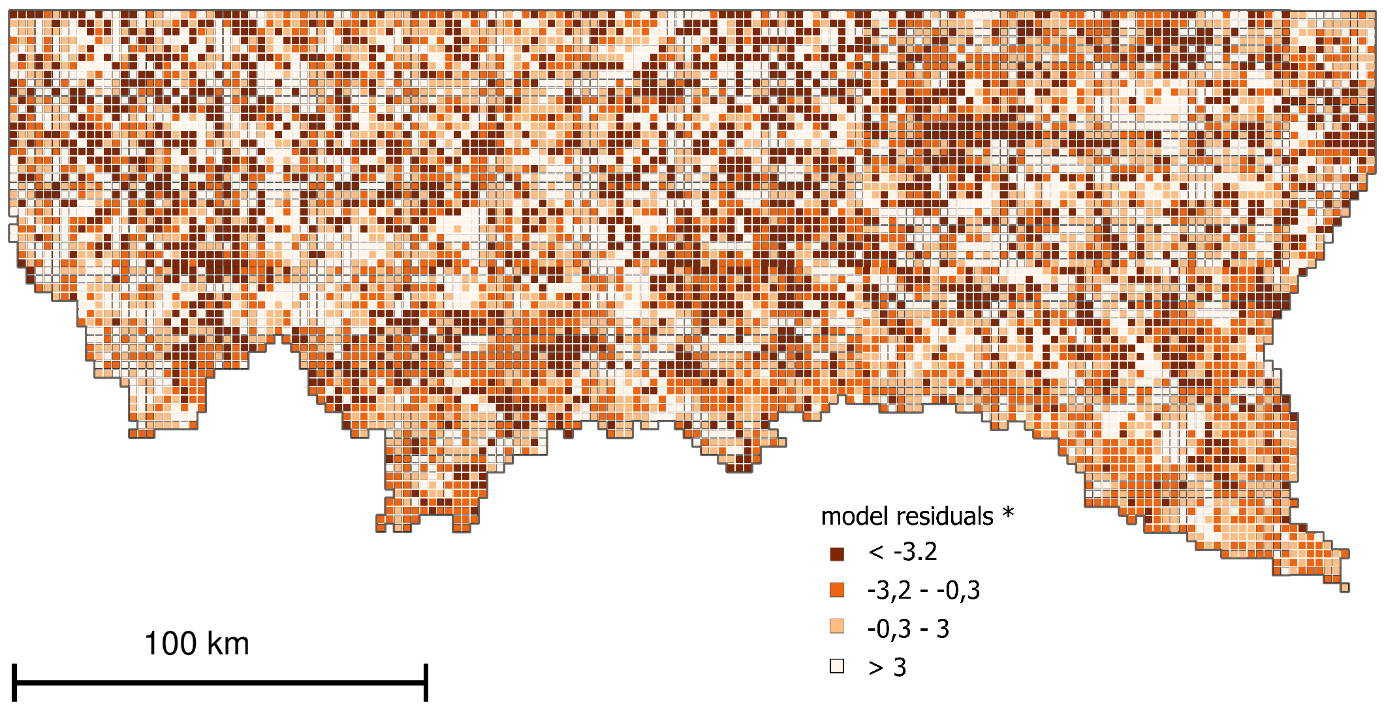


Fig. S1. The values of AUC for boosted regression trees models with excluded particular percentage of potentially undersampled squares. For final modelling selected was ban of 25% of squares (rectangle).


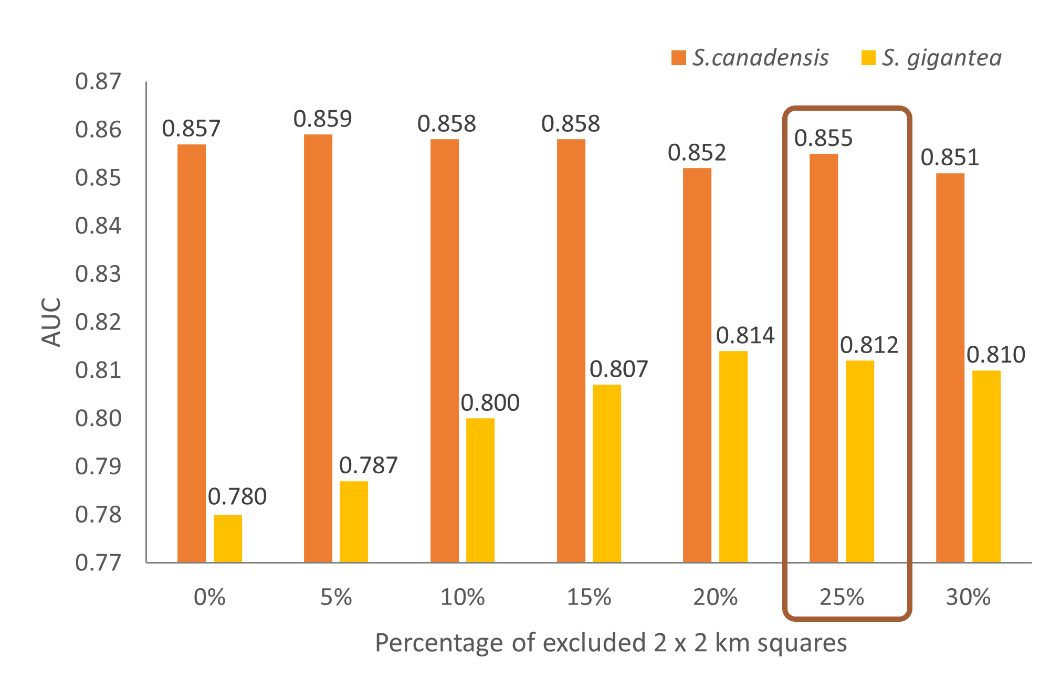


Barbet‐Massin, M., Jiguet, F., Albert, C. H., & Thuiller, W. (2012). Selecting pseudo‐absences for species distribution models: how, where and how many?. *Methods in Ecology and Evolution*, 3(2), 327-338.

Elith, J., Graham, C., Valavi, R., Abegg, M., Bruce, C., Ford, A., ... & Loiselle, B. (2020). Presence-only and Presence-absence Data for Comparing Species Distribution Modeling Methods. *Biodiversity Informatics*, 15(2), 69-80.

MacKenzie, D. I., & Royle, J. A. (2005). Designing occupancy studies: general advice and allocating survey effort. *Journal of Applied Ecology*, 42(6), 1105-1114.

Ponder, W. F., Carter, G. A., Flemons, P., & Chapman, R. R. (2001). Evaluation of museum collection data for use in biodiversity assessment. *Conservation Biology*, 15(3), 648-657.

Szymura T. H., Szymura M., Zając M., & Zając A. (2018). Effect of anthropogenic factors, landscape structure, land relief, soil and climate on risk of alien plant invasion at regional scale. *Science of The Total Environment*, 626, 1373-1381. https://doi.org/10.1016/j.scitotenv.2018.01.131

Zając, A., & Zając, M., (2015). Distribution of Kenophytes in the Polish Carpathians and their Foreland. –Instytut Botaniki Uniwersytetu Jagiellońskiego, Kraków, 304.

2. Table S1. Explanatory variables used for modelling. Descriptions, abbreviations and data sources.

| **Variable name** | **Abbreviation** | **Units** | **Description** | **Data source** |
| --- | --- | --- | --- | --- |
| Communication routes (railways and roads) density | communication | km | Sum of roads and railways length in square | Calculated basing on BDOO data |
| Human population density | density | N/km^2^ | Human population density in 1km^2^ grid, basing on residents register data | Central Statistical Office of Poland |
| Income per capita | income | zl per capita | Total annual income per capita in basic administrative unit (gmina) |  |
| Urban area percentage | urban | % | Percentage of urbanized areas | Calculated basing on CORINE 2012 |
| Shannon’s diverstiy index of landscape | SHDI | dimensionless | Shannon's diversity index of the landscape. SHDI equals minus the sum, across all patch types, of the proportional abundance of each patch type multiplied by that proportion. Highest values of SHDI denote higher landscape diversity. |  |
| Cropland area percentage | cropland | % | Percentage of croplands area in square (CLC classes 211-223, 241-244) |  |
| Forest area percentage | forest | % | Percentage of forest area in square (CLC class 311 and 313) |  |
| Topographic roughness index | TRI | dimensionless, high values reflect high variability of altitude | Reflects local differentiation of altitude (Hengl & Reuter 2008). | Calculated basing on DEM^#^ |
| Topographic position index | TPI | dimensionless – high value reflects convex form, while low – concave. | Compare elevation of central rasters with peripherial within a buffers (DeReu 2013). |  |
| Topographic wetness index | TWI | dimensionless, high values reflect potentially moister soil, while low values – dry areas | The TWI modelled accumulation of water resulting from location within the catchment, the catchment size and slope (Hengl & Reuter 2008). |  |
| Average annual temperature | temperature | °C | annual mean temperature | Climatic model (Hijmans et al., 2005) |
| Temperature seasonality | Ts | °C | standard deviation of monthly temperature averages in a year multiplied by 100 |  |
| Annual sum of precipitation | precipitation | mm | annual sum of precipitation |  |
| CaCO_3_ content | CaCO_3_ | g*kg^-1^ | The value for topsoil | Maps basing on LUCAS database (Ballabio et al., 2019) |
| K content | K | mg*kg^-1^ |  |  |
| N content | N | g*kg^-1^ |  |  |
| P content | P | mg*kg^-1^ |  |  |
| pH in H_2_O | pH | pH scale |  |  |
| Distance to nearest introduction site *S. Canadensis* | distance_S.can | km | Distance from each 2x2 km square to nearest site with confirmed presence of particular *Solidago* species in 1950s | Calculated basing on map Tokarska-Guzik (2005) |
| Distance to nearest introduction site *S. gigantean* | distance_S.gig | km |  |  |

^#^Calculations of secondary topographic features were done basing on digital elevation model (DEM) in 30x30 m grid obtained from GMES RDA project: https://www.eea.europa.eu/data-and-maps/data/eu-dem

Ballabio, C., Lugato, E., Fernández-Ugalde, O., Orgiazzi, A., Jones, A., Borrelli, P., ... & Panagos, P. (2019). Mapping LUCAS topsoil chemical properties at European scale using Gaussian process regression. *Geoderma*, 355, 113912.

Baza danych obiektów ogólnogeograficznych (BDOO) http://www.codgik.gov.pl/index.php/zasob/baza-danych-ogolnogeograficznych.html

Central Statistical Office of Poland: http://stat.gov.pl/en/

Corine Land Cover http://clc.gios.gov.pl/

De Reu, J., Bourgeois, J., Bats, M., Zwertvaegher, A., Gelorini, V., De Smedt, P., ... & Crombé, P. (2013). Application of the topographic position index to heterogeneous landscapes. Geomorphology, 186, 39-49.Hengl T, Reuter HI, editors. Geomorphometry: Concepts, Software, Applications. Developments in Soil Science. 33rd ed. Elsevier; 2008.

Hijmans, R. J., Cameron, S. E., Parra, J. L., Jones, P. G., & Jarvis, A. (2005). Very high resolution interpolated climate surfaces for global land areas. *International journal of climatology*, 25(15), 1965-1978.

Tokarska-Guzik, B. (2005). The establishment and spread of alien plant species (kenophytes) in the flora of Poland. Katowice: Wydawnictwo Uniwersytetu Śląskiego.

3. Tab. S2. Descriptive statistics of environmental variables. Variables used in modelling are distinguished by bold. To avoid collinearity, variables correlate equal or above the absolute value of Pearson correlation coefficient 0.7 were excluded from modelling (Dormann et al., 2013). The correlation matrix of environmental variables is shown in Tab. S3.

| **variable** | **average** | **st. dev.** | **min.** | **max.** | **median** | **Q25** | **Q75** |
| --- | --- | --- | --- | --- | --- | --- | --- |
| **communication** | 2.75 | 2.39 | 0.00 | 26.10 | 2.40 | 0.80 | 4.00 |
| **SHDI** | 0.79 | 0.35 | 0.00 | 1.74 | 0.80 | 0.57 | 1.05 |
| urban | 10.01 | 16.87 | 0.00 | 100.00 | 1.60 | 0.00 | 14.00 |
| **cropland** | 44.93 | 30.91 | 0.00 | 100.00 | 47.20 | 15.90 | 71.30 |
| forest | 35.70 | 33.11 | 0.00 | 100.10 | 27.60 | 3.10 | 62.40 |
| **density** | 245.69 | 684.29 | 0.00 | 13180.00 | 103.00 | 36.00 | 198.00 |
| **income** | 3213.43 | 587.22 | 2277.00 | 5706.00 | 3067.20 | 2830.20 | 3469.00 |
| TRI | 3.29 | 2.68 | 0.15 | 20.69 | 2.73 | 1.02 | 4.72 |
| **TPI** | 1.88 | 67.92 | -238.60 | 621.50 | -5.60 | -28.00 | 21.00 |
| **temperature** | 7.23 | 1.15 | -0.50 | 8.80 | 7.50 | 6.50 | 8.10 |
| **TWI** | 8.83 | 1.24 | 6.06 | 16.06 | 8.59 | 7.84 | 9.75 |
| **Ts** | 777.76 | 31.29 | 597.00 | 845.00 | 778.00 | 755.00 | 804.00 |
| precipitation | 744.07 | 115.66 | 586.30 | 1632.50 | 713.80 | 671.80 | 791.00 |
| **CaO_3_** | 6.87 | 8.61 | 0.00 | 67.92 | 3.86 | 1.50 | 8.66 |
| **K** | 135.67 | 38.79 | 42.19 | 318.18 | 131.75 | 107.12 | 158.74 |
| N | 2.07 | 0.59 | 0.85 | 5.06 | 1.92 | 1.64 | 2.43 |
| P | 23.95 | 8.34 | 4.90 | 47.21 | 23.14 | 16.93 | 31.16 |
| **pH** | 5.56 | 0.46 | 4.20 | 6.80 | 5.61 | 5.22 | 5.90 |
| **distance_S.can** | 56.67 | 27.77 | 0.00 | 140.00 | 56.00 | 35.00 | 78.00 |
| **distance_S.gig** | 47.12 | 32.02 | 0.00 | 170.00 | 41.00 | 23.00 | 64.00 |

Dormann, C. F., Elith, J., Bacher, S., Buchmann, C., Carl, G., Carré, G., ... & Lautenbach, S. (2013). Collinearity: a review of methods to deal with it and a simulation study evaluating their performance. *Ecography*, *36*(1), 27-46.

4. Tab. S3. Correlation matrix between environmental variables. Pearson correlation coefficients equal or above absolute value of 0.7 are distinguished by grey.

|  | communi-cation | SHDI | urban | cropland | forest | density | income | TRI | TPI | tempera-ture | TWI | Ts | precipita-tion | CaCO_3_ | K | N | P | pH | distance_  S.can | distance_  S.gig |
| --- | --- | --- | --- | --- | --- | --- | --- | --- | --- | --- | --- | --- | --- | --- | --- | --- | --- | --- | --- | --- |
| communication |  | 0.064 | 0.670 | 0.205 | -0.515 | 0.537 | 0.063 | -0.417 | -0.347 | 0.465 | 0.397 | 0.153 | -0.285 | -0.042 | 0.394 | -0.304 | 0.468 | 0.419 | -0.177 | -0.300 |
| SHDI | 0.064 |  | 0.076 | -0.258 | 0.037 | -0.087 | -0.055 | -0.075 | -0.205 | 0.120 | 0.124 | 0.016 | -0.016 | -0.017 | -0.108 | -0.124 | -0.052 | -0.190 | -0.013 | -0.049 |
| urban | 0.670 | 0.076 |  | -0.078 | -0.417 | 0.733 | 0.203 | -0.349 | -0.228 | 0.359 | 0.369 | 0.045 | -0.171 | 0.023 | 0.374 | -0.215 | 0.416 | 0.354 | -0.091 | -0.254 |
| cropland | 0.205 | -0.258 | -0.078 |  | -0.777 | -0.113 | -0.340 | -0.451 | -0.314 | 0.455 | 0.304 | 0.337 | -0.383 | -0.134 | 0.410 | -0.370 | 0.566 | 0.605 | -0.375 | -0.281 |
| forest | -0.515 | 0.037 | -0.417 | -0.777 |  | -0.244 | 0.180 | 0.625 | 0.431 | -0.581 | -0.585 | -0.291 | 0.407 | 0.045 | -0.523 | 0.403 | -0.776 | -0.790 | 0.373 | 0.351 |
| density | 0.537 | -0.087 | 0.733 | -0.113 | -0.244 |  | 0.261 | -0.198 | -0.118 | 0.223 | 0.187 | 0.002 | -0.098 | 0.028 | 0.269 | -0.136 | 0.230 | 0.238 | -0.067 | -0.196 |
| income | 0.063 | -0.055 | 0.203 | -0.340 | 0.180 | 0.261 |  | 0.141 | 0.038 | -0.168 | -0.071 | -0.157 | 0.105 | 0.029 | -0.083 | 0.217 | -0.148 | -0.103 | 0.262 | 0.087 |
| TRI | -0.417 | -0.075 | -0.349 | -0.451 | 0.625 | -0.198 | 0.141 |  | 0.429 | -0.798 | -0.822 | -0.531 | 0.755 | 0.386 | -0.221 | 0.737 | -0.724 | -0.326 | 0.409 | 0.330 |
| TPI | -0.347 | -0.205 | -0.228 | -0.314 | 0.431 | -0.118 | 0.038 | 0.429 |  | -0.467 | -0.427 | -0.305 | 0.353 | 0.182 | -0.184 | 0.262 | -0.297 | -0.248 | 0.087 | 0.076 |
| temperature | 0.465 | 0.120 | 0.359 | 0.455 | -0.581 | 0.223 | -0.168 | -0.798 | -0.467 |  | 0.692 | 0.458 | -0.725 | -0.286 | 0.448 | -0.757 | 0.705 | 0.354 | -0.457 | -0.596 |
| TWI | 0.397 | 0.124 | 0.369 | 0.304 | -0.585 | 0.187 | -0.071 | -0.822 | -0.427 | 0.692 |  | 0.364 | -0.567 | -0.138 | 0.177 | -0.498 | 0.715 | 0.400 | -0.311 | -0.324 |
| Ts | 0.153 | 0.016 | 0.045 | 0.337 | -0.291 | 0.002 | -0.157 | -0.531 | -0.305 | 0.458 | 0.364 |  | -0.797 | -0.519 | -0.039 | -0.523 | 0.311 | 0.023 | -0.341 | 0.057 |
| precipitation | -0.285 | -0.016 | -0.171 | -0.383 | 0.407 | -0.098 | 0.105 | 0.755 | 0.353 | -0.725 | -0.567 | -0.797 |  | 0.510 | -0.164 | 0.717 | -0.551 | -0.143 | 0.504 | 0.181 |
| CaCO_3_ | -0.042 | -0.017 | 0.023 | -0.134 | 0.045 | 0.028 | 0.029 | 0.386 | 0.182 | -0.286 | -0.138 | -0.519 | 0.510 |  | 0.125 | 0.392 | -0.084 | 0.303 | 0.074 | -0.094 |
| K | 0.394 | -0.108 | 0.374 | 0.410 | -0.523 | 0.269 | -0.083 | -0.221 | -0.184 | 0.448 | 0.177 | -0.039 | -0.164 | 0.125 |  | -0.240 | 0.524 | 0.588 | -0.292 | -0.416 |
| N | -0.304 | -0.124 | -0.215 | -0.370 | 0.403 | -0.136 | 0.217 | 0.737 | 0.262 | -0.757 | -0.498 | -0.523 | 0.717 | 0.392 | -0.240 |  | -0.564 | -0.092 | 0.506 | 0.429 |
| P | 0.468 | -0.052 | 0.416 | 0.566 | -0.776 | 0.230 | -0.148 | -0.724 | -0.297 | 0.705 | 0.715 | 0.311 | -0.551 | -0.084 | 0.524 | -0.564 |  | 0.689 | -0.479 | -0.426 |
| pH | 0.419 | -0.190 | 0.354 | 0.605 | -0.790 | 0.238 | -0.103 | -0.326 | -0.248 | 0.354 | 0.400 | 0.023 | -0.143 | 0.303 | 0.588 | -0.092 | 0.689 |  | -0.251 | -0.276 |
| distance_S.can | -0.177 | -0.013 | -0.091 | -0.375 | 0.373 | -0.067 | 0.262 | 0.409 | 0.087 | -0.457 | -0.311 | -0.341 | 0.504 | 0.074 | -0.292 | 0.506 | -0.479 | -0.251 |  | 0.479 |
| distance_S.gig | -0.300 | -0.049 | -0.254 | -0.281 | 0.351 | -0.196 | 0.087 | 0.330 | 0.076 | -0.596 | -0.324 | 0.057 | 0.181 | -0.094 | -0.416 | 0.429 | -0.426 | -0.276 | 0.479 |  |

5. Map S2. Distribution of goldenrods (black squares) in 1950s and distances to the sites. Maps prepared on the basis Tokarska-Guzik (2005).


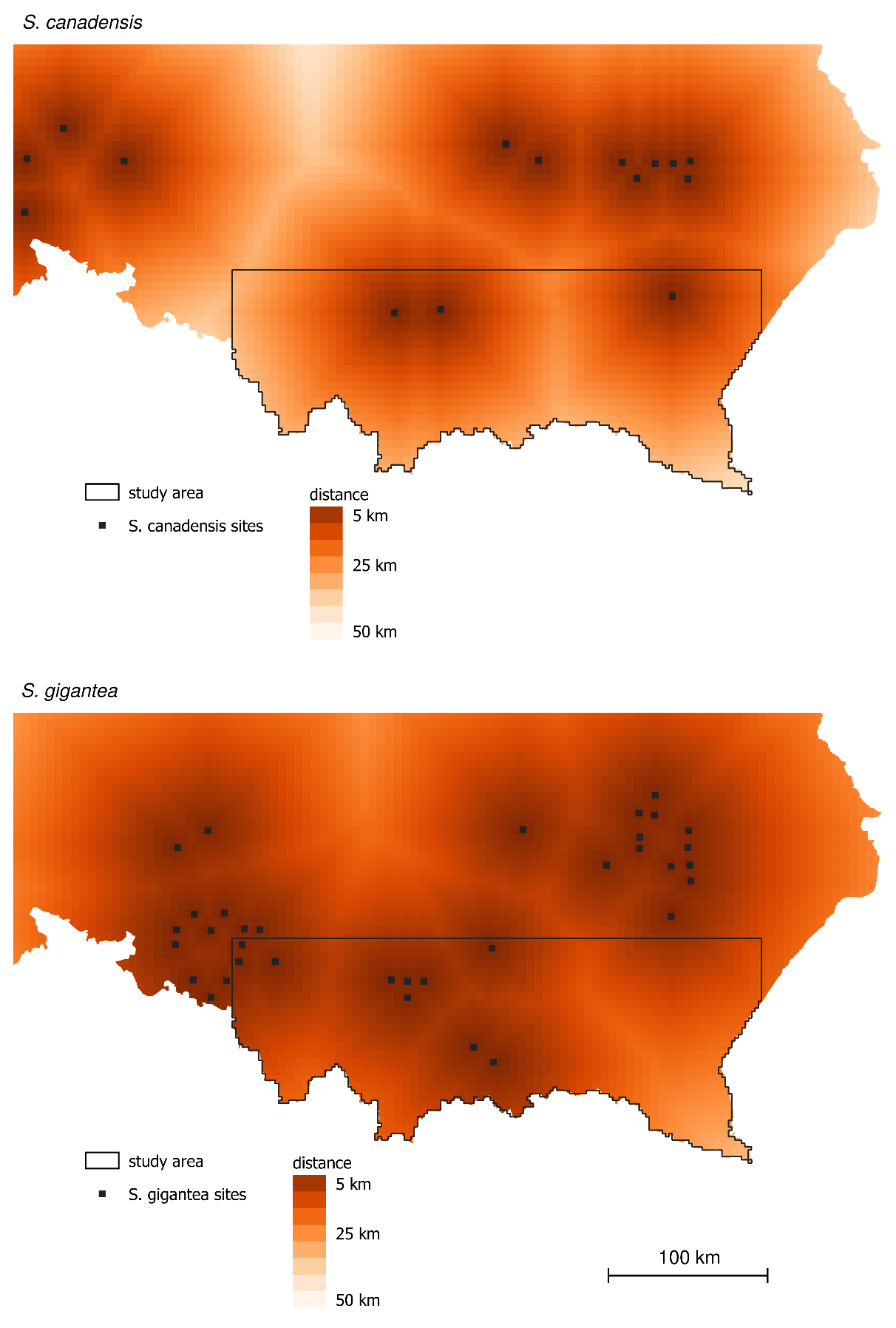


Tokarska-Guzik, B. (2005). The establishment and spread of alien plant species (kenophytes) in the flora of Poland. Katowice: Wydawnictwo Uniwersytetu Śląskiego.

6. Map S3. Spatial blocks used for cross-validation models. The size of block were: 20 x 20 km for *S. canadensis* and 10 x 10 km for *S. gigantea*. The arrangement of blocks into optimal cross-validation folds was set according to random pattern basing on 9999 iterations (Roberts et al., 2017; Valavi et al., 2019).


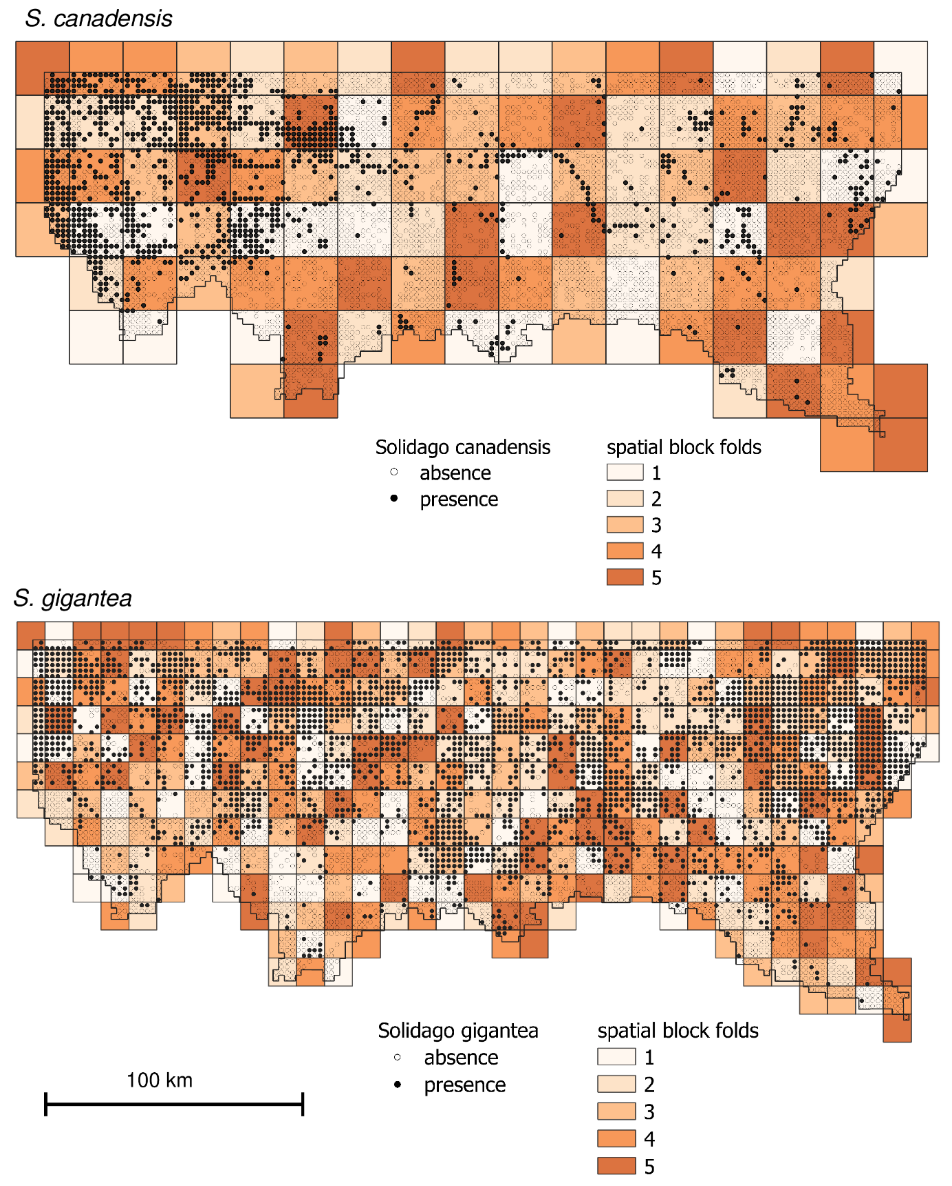


Roberts, D. R., Bahn, V., Ciuti, S., Boyce, M. S., Elith, J., Guillera‐Arroita, G., ... & Warton, D. I. (2017). Cross‐validation strategies for data with temporal, spatial, hierarchical, or phylogenetic structure. Ecography, 40(8), 913-929.

Valavi, R., Elith, J., Lahoz‐Monfort, J. J., & Guillera‐Arroita, G. (2019). block CV: An r package for generating spatially or environmentally separated folds for k‐fold cross‐validation of species distribution models. Methods in Ecology and Evolution, 10(2), 225-232.

7. Map S4. Comparison of predicted with observed distribution of goldenrods. Squares projected to be suitable, but not yet colonized are marked by orange for a) *S. canadensis*, b) *S. gigantea*, and c) both species. Squares considered as suitable were those for which the probability of species occurrence exceeded the optimal cutoff values.


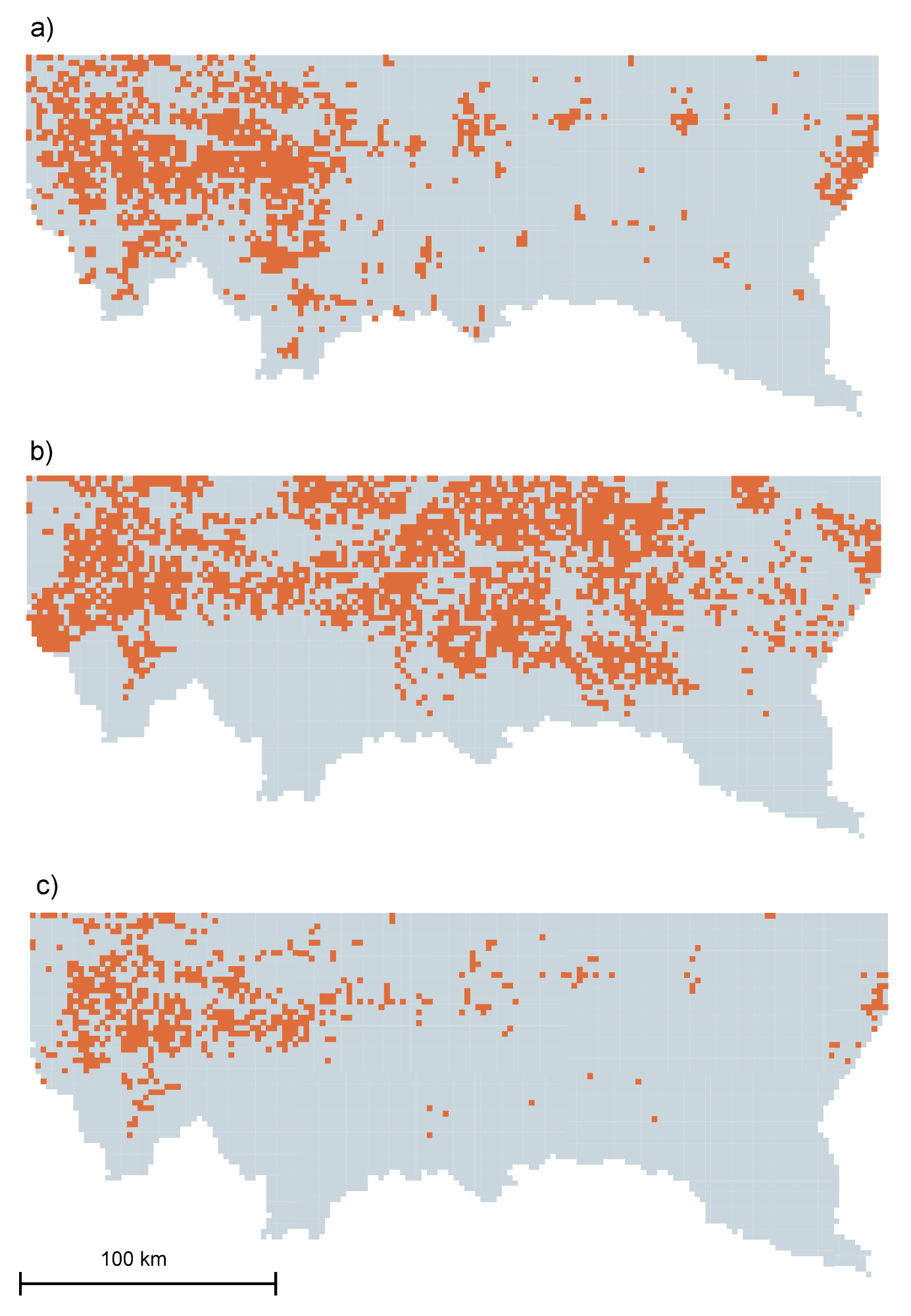


Drivers of Solidago species invasion in Central Europe—Case study in the landscape of the Carpathian Mountains and their foreground

– ODMAP Protocol –

Perera P.C.D, Szymura T.H., Chmolowska D., Zajac A, Szymura M.

2021-02-14

## Overview

#### Authorship

Contact: tomasz.szymura@uwr.edu.pl

Study link: under review

#### Model objective

Model objective: Inference and explanation

#### Focal Taxon

Focal Taxon: *Solidago canadensis*, *Solidago gigantea*

#### Location

Location: Polish part of Carpathian Mts and its foreground

#### Scale of Analysis

Spatial extent: 18.59, 23.06, 49, 50.27 (xmin, xmax, ymin, ymax)

Spatial resolution: 320 x 130 km (area: ca 31.200 km2)

Temporal extent: single time slice modelling, climate data for 1970-2000, the data on invasive *Solidago* distribution were derived from atlas published in 2015

Boundary: political, rectangle

#### Biodiversity data

Observation type: field survey, range map

Response data type: presence/absence

#### Predictors

Predictor types: climatic, habitat, edaphic, topographic

#### Hypotheses

Hypotheses: we searched for crucial invasion drivers within the PAB (propagule pressure, abiotic factor, biotic interactions) framework

#### Assumptions

Model assumptions: we assume that both species are in quasi-equilibrium with environment

#### Algorithms

Modelling techniques: brt

Model complexity: ability to provide parsimonious model through model simplification procedure

Model averaging: averaging of 5 models created with 5-fold spatially blocked cross-validation

#### Workflow

Model workflow: 1. Ban of potentially undersampled squares based on ‘target species group’ approach and established previously models 2. Tuning the BRT setting 3. Model simplification 4. Spatially blocked cross validation of simplified models

#### Software

Software: biomod2, brt, gbm packages in R environment

Code availability: standard codes

Data availability: All data are available publicly from the sources given in the manuscript.

## Data

#### Biodiversity data

Taxon names: *Solidago canadensis, Solidago gigantea*

Taxonomic reference system: -

Ecological level: species

Data sources: Zając, A., & Zając, M., (2015). Distribution of Kenophytes in the Polish Carpathians and their Foreland. –Instytut Botaniki Uniwersytetu Jagiellońskiego, Kraków

Sampling design: uniform

Sample size: *S. canadensis*: 1255 presences/4595 absences. *S. gigantea*: 3107 presences/2743absences

Clipping: Central Europe, Poland

Scaling: -

Cleaning: -

Absence data: -

Background data: -

Errors and biases: potentially undersampled 2 x 2 km squares removed prior to analysis

#### Predictor variables

Predictor variables: 21 predictor, being the proxies of propagule pressure, abiotic and biotic factors (PAB framework)

Data sources: All data are available publicly from the sources given in the manuscript.

Spatial extent: 18.59, 23.06, 49.00, 50.27 (xmin, xmax, ymin, ymax)

Spatial resolution: 2 x 2 km grid

Coordinate reference system: EPSG: 2190

Temporal extent: -

#### Transfer data

Spatial extent: 18.59, 23.06, 49.00, 50.27 (xmin, xmax, ymin, ymax)

Spatial resolution: 2 x 2 km

Temporal extent: single time slice, current for explanatory variables

## Model

#### Multicollinearity

Multicollinearity: Exclusion of one variable if correlation coefficient > 0.7

#### Model settings

brt: distribution (bernoulli), nTrees (3850-3900), interactionDepth (5), shrinkage (0.001), bagFraction (0.5), trainFraction (10 -fold corss-validation)

#### Model estimates

Coefficients: average for 5-fold spatially blocked cross-validation

#### Analysis and Correction of non-independence

Spatial autocorrelation: No

Temporal autocorrelation: No

Nested data: No

## Assessment

#### Performance statistics

Performance on training data: AUC

#### Plausibility check

Response shapes: evaluation streeps, variables importance
